# Efficacy and Toxicity of Whole Pelvic Radiotherapy Versus Prostate-Only Radiotherapy in Localized Prostate Cancer: A Systematic Review and Meta-Analysis

**DOI:** 10.3389/fonc.2021.796907

**Published:** 2022-01-27

**Authors:** Shilin Wang, Wen Tang, Huanli Luo, Fu Jin, Ying Wang

**Affiliations:** ^1^ Department of Radiation Oncology, Chongqing University Cancer Hospital & Chongqing Cancer Institute & Chongqing Cancer Hospital, Chongqing, China; ^2^ Department of Rehabilitation, The Second Affiliated Hospital of Chongqing Medical University, Chongqing, China

**Keywords:** localized prostate cancer, meta-analysis, whole pelvic radiotherapy, survival, genitourinary toxicity (GU)

## Abstract

**Background:**

There is little level 1 evidence regarding the relative efficacy and toxicity of whole pelvic radiotherapy (WPRT) compared with prostate-only radiotherapy (PORT) for localized prostate cancer.

**Methods:**

We used Cochrane, PubMed, Embase, Medline databases, and ClinicalTrials.gov to systematically search for all relevant clinical studies. The data on efficacy and toxicity were extracted for quality assessment and meta-analysis to quantify the effect of WPRT on biochemical failure-free survival (BFFS), progression-free survival (PFS), distant metastasis-free survival (DMFS), overall survival (OS), gastrointestinal (GI) toxicity, and genitourinary (GU) toxicity compared with PORT. The review is registered on PROSPERO, number: CRD42021254752.

**Results:**

The results revealed that compared with PORT, WPRT significantly improved 5-year BFFS and PFS, and it was irrelevant to whether the patients had undergone radical prostatectomy (RP). In addition, for the patients who did not receive RP, the 5-year DMFS of WPRT was better than that of PORT. However, WPRT significantly increased not only the grade 2 or worse (G2+) acute GI toxicity of non-RP studies and RP studies, but also the G2+ late GI toxicity of non-RP studies. Subgroup analysis of non-RP studies found that, when the pelvic radiation dose was >49 Gy (equivalent-doses-in-2-Gy-fractions, EQD-2), WPRT was more beneficial to PFS than PORT, but significantly increased the risk of G2+ acute and late GU toxicity.

**Conclusions:**

Meta-analysis demonstrates that WPRT can significantly improve BFFS and PFS for localized prostate cancer than PORT, but the increased risk of G2+ acute and late GI toxicity must be considered.

**Systematic Review Registration:**

PROSPERO CRD42021254752.

## Background

Prostate cancer is the most frequent cancer in men, accounting for more than 1 in 5 new diagnoses (1, 2). In western societies, prostate cancer has a high cure rate, but it is also the second leading cause of cancer deaths for men (3, 4). The main treatments for prostate cancer are radical prostatectomy (RP), radiotherapy (RT), and hormone therapy (HT). RT is a crucial treatment strategy for men who received a diagnosis of localized prostate cancer (5, 6). However, there has been considerable controversy over whether to choose whole pelvic radiotherapy (WPRT) or prostate-only radiotherapy (PORT) for localized prostate cancer.

At present, there is little level 1 evidence regarding the relative efficacy and toxicity of WPRT compared with PORT for localized prostate cancer, and relevant clinical randomized controlled trials have drawn confusing conclusions (7–12). A Phase III clinical trial, the Radiation Therapy Oncology Group (RTOG) 9413, showed that for localized prostate cancer, WPRT improved progression-free survival (PFS) compared with PORT (12–14). However, the French Genitourinary Study Group (GETUG)-01 found that compared with PORT, WPRT had no statistically significant improvement in PFS, event-free survival (EFS), and overall survival (OS) (11, 15). A recent Phase III randomized clinical trial of prostate-only or whole-pelvic radiation therapy in high-risk prostate cancer (POP-RT) pointed out that WPRT for localized prostate cancer improved the biochemical failure-free survival (BFFS) and distant metastasis-free survival (DMFS) compared with PORT, but resulted in a significant increase in late grade 2 or worse (G2+) genitourinary (GU) toxicity (9, 16).

This study systematically reviewed clinical studies comparing WPRT to PORT for localized prostate cancer. The data on efficacy and toxicity were extracted for quality assessment and meta-analysis to quantify the effect of WPRT on BFFS, PFS and DMFS, OS, gastrointestinal (GI) toxicity, and GU toxicity compared with PORT.

## Methods

### Literature Search

We used Cochrane, PubMed, Embase, Medline databases, and ClinicalTrials.gov to systematically search for eligible trials from inception until October 10, 2021, by two study investigators independently. The following search terms were used: “Prostatic Neoplasms”[Mesh] AND “Radiotherapy”[Mesh] AND “Pelvis”[Mesh]. Detailed search terms were shown in **Supplementary Table 1**. The review is registered on PROSPERO, number: CRD42021254752.

### Inclusion Criteria, Study Eligibility, and Data Extraction

**Figure 1:**
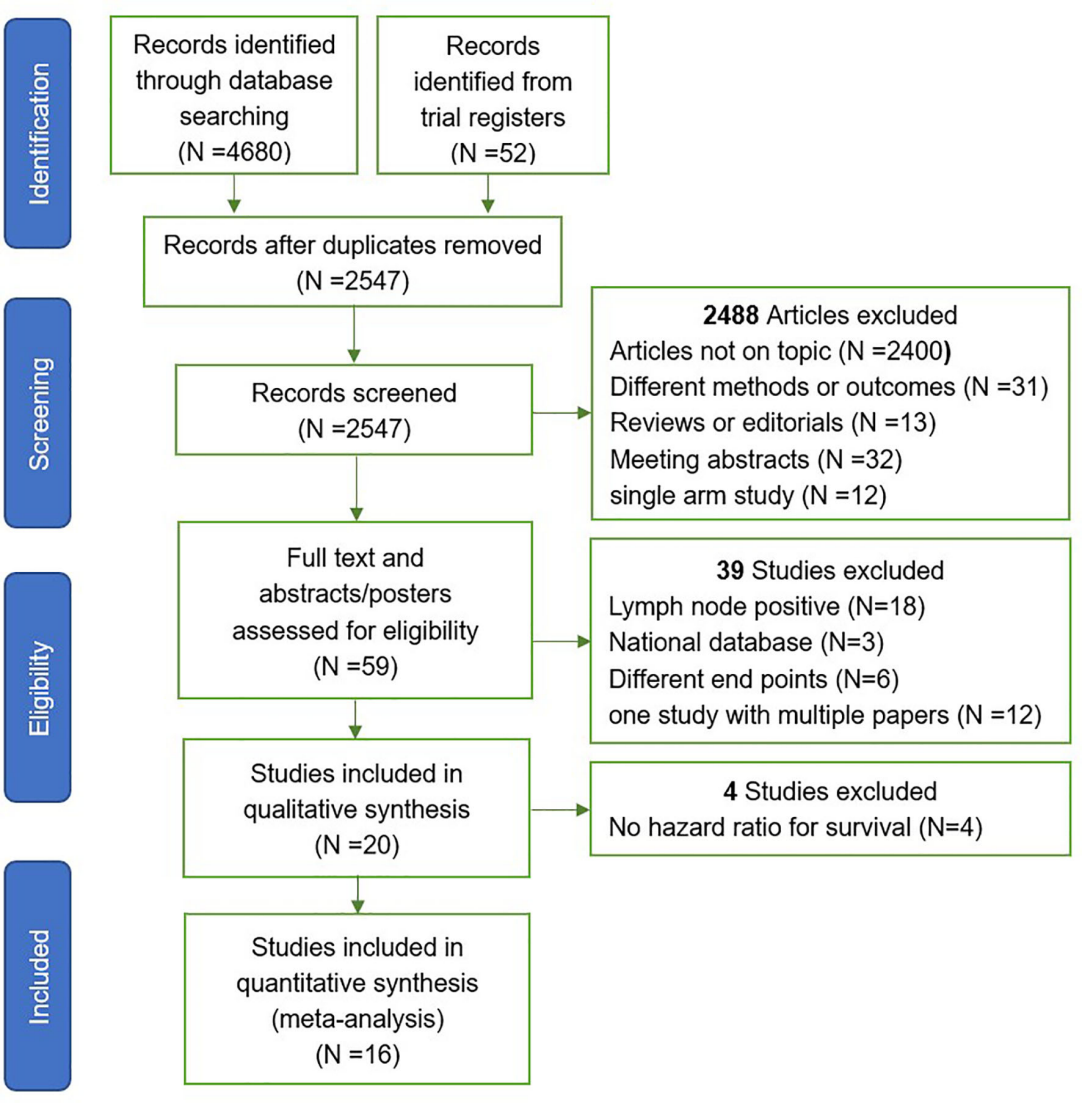
Study selection. Flowchart of trials included and excluded from meta-analysis.

The Preferred Reporting Items for Systematic Reviews and Meta-analysis (PRISMA) criteria were used for article selection (**Figure 1**). Two investigators independently searched and selected literature, included in randomized controlled trials (RCTs) and cohort studies (CRS), and excluded articles with metastatic prostate cancer. The article must include one of the biochemical failure-free survival (BFFS), progression free survival (PFS), distant metastasis-free survival (DMFS), survival overall (OS), gastrointestinal (GI) toxicity, and genitourinary (GU) toxicity for data extraction. For studies with multiple publications, or where there was overlap in the patients studied, the most recent publication was chosen. Any queries were checked by a second reviewer and resolved by consensus.

### Statistical Analysis

The results of time-to-event outcomes, BFFS, PFS, DMFS, and OS, were reported as hazard ratios (HRs) with 95% confidence interval (CIs), and the most fully adjusted HRs were extracted to prevent interference from other variables. The results of GI toxicity and GU toxicity were recorded as risk ratios (RRs) with 95% CIs. The following effect modifiers on the end points were tested using subgroup analysis: radical prostatectomy (RP), androgen deprivation therapy (ADT), radiation dose, and radiotherapy technology.

Heterogeneity was assessed using the *χ* ^2^ test and the *I* ^2^ statistic. Significant heterogeneity was indicated by *p* < 0.05 in Cochrane Q tests and a ratio greater than 50% in *I* ^2^ statistics, which led to the use of random-effects models according to the DerSimonian and Laird method (17, 18). Otherwise, these tests were negative for heterogeneity, and fixed-effects models were chosen. Statistical analyses were performed using the Cochrane Review Manager, version 5.3. A confidence level of 95% (*p* < 0.05) was considered statistically significant.

### Risk of Bias

RCTs were evaluated using the Cochrane Risk of Bias Tool (19), and the CRS were analyzed using the Newcastle-Ottawa Scale (20). Furthermore, funnel plots of standard errors vs. effect estimates were inspected for publication bias (21, 22).

## Results

### Included Studies

A total of 4,680 publications and 52 registered clinical studies were identified from the literature search, and 16 studies that met the eligibility criteria were finally selected, including 6 randomized controlled trials (RCTs) (7–12) and 10 cohort studies (CRS) (23–32). Four studies that met the inclusion criteria were excluded because of no hazard ratio available for survival (33–36). The PRISMA study selection diagram is shown in **Figure 1**. Of the 16 studies, 9 studies did not perform radical prostatectomy (non-RP), and the patients of the other 7 studies received radical prostatectomy (RP). A total of 10,212 participants were enrolled, of which 3,393 participants received whole pelvic radiotherapy (WPRT), and 6,819 participants received prostate-only radiotherapy (PORT). The overall median follow-up time for efficacy was 64.8 months, and toxicity was 50.5 months. A summary of the studies characteristics is presented in **Table 1**.

**Table 1:** Studies characteristics.

Author (year or trial name)	Patient characteristics			Follow-up(median)	Sample		Radiation Dose*		Other therapies	Outcome		
	Inclusion criteria	Radiotherapy	Country		WP	PO	Prostate	Pelvis		Survival	GI	GU
Blanchard et al. (GETUG 12) (7)	High-risk localized, lymphadenectomy	3D-CRT	France	8.8 years	208	150	74–78	48	ADT	PFS	X	✓
Braunstein et al. (2015) (23)	T1c-T3, N0, M0	NR	USA	3.3 years	486	2237	NR	44.25	ADT	OS	X	X
Dearnaley et al. (PIVOTAL) (8)	Localized, T3b/T4	IMRT	UK	37.6 months	60	62	74	58.1	ADT	X	✓	X
Deville et al. (2011) (24)	GS>7, T2a-c	IMRT	USA	25 months	36	31	70	44.25	RP/ADT	X	✓	✓
Ishii et al. (2017) (25)	Localized	Arc	Japan	24 months	126	108	78	46.02	ADT	X	✓	✓
Link et al. (2019) (26)	GS >6, Localized	IMRT/Arc	Germany	62.2 months	43	77	79/71	44	RP/ADT	BFFS	X	X
Mantini et al. (2011) (27)	Localized	3D-CRT	Italy	52 months	168	190	70/74	44.25	ADT	X	✓	✓
McDonald et al. (2014) (28)	Localized	Tomo/Arc/IMRT	USA	4 years	103	109	72.92	49.56	ADT	X	✓	✓
Moghanaki et al. (2012) (29)	GS≥8,Lymph node-negative	3D-CRT	USA	48.5 months	112	135	59–74	49.56	RP/ADT	BFFS	X	X
Murthy et al. (POP-RT) (9)	Lymph nodal risk≥20%, localized	IMRT	India	68 months	110	114	72.08	50	ADT	BFFS, DMFS, OS	✓	✓
Pollack et al. (RTOG 0534) (10)	GS ≤ 9,T2-T3N0/Nx	3D-CRT/IMRT	USA	5.4 years	574	578	63.92–68.83	44.25	RP/ADT	BFFS, PFS, DMFS, OS	X	X
Pommier et al. (GETUG-01) (11)	T1b-T3, cN0pNx, localized	FFRT/3D-CRT	France	11.4 years	225	221	66–70	46	ADT	PFS	✓	✓
Ramey et al. (2017) (30)	pT1-4, Nx/0, cM0,GS≥8	2D-RT/3D-CRT/IMRT	USA	51 months	245	1616	66	NR	RP/ADT	BFFS, DMFS	X	X
Roach et al. (RTOG 9413) (12)	Lymph nodal risk≥15%, GS>6, localized	3D-CRT	USA	8.8 years	661	661	69.03	49.56	ADT	PFS	✓	✓
Song et al. (2019) (31)	Lymph node-negative	3D-CRT/IMRT	Korea	66 months	108	83	66	46	RP/ADT	BFFS	X	X
Waldstein et al. (2017) (32)	GS≥8, node-negative	3D-CRT	Austria	49 months	128	447	67	47.5	RP/ADT	X	✓	✓

3D-CRT, three-dimensional conformal radiotherapy; 2D-RT, two-dimensional radiotherapy; FFRT, four-field box radiotherapy; IMRT, intensity-modulated radiation therapy; Arc, volumetric modulated arc therapy; Tomo, tomotherapy; WP, whole-pelvic radiotherapy; PO, prostate-only radiotherapy; GS, Gleason score; RP, radical prostatectomy; ADT, androgen deprivation therapy; BT, brachytherapy; OS, overall survival; BFFS, biochemical failure-free survival; PFS, progression-free survival; DMFS, Distant metastasis-free survival, GI, gastrointestinal; GU, genitourinary; NR, not reported.

*Equivalent-doses-in-2-Gy-fractions, EQD-2.

### Biochemical Failure-Free Survival

**Figure 2:**
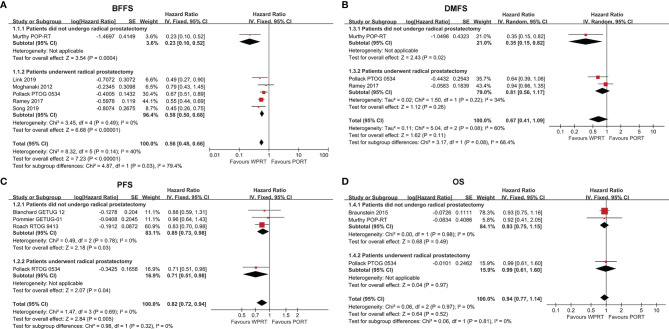
Forest plots of BFFS **(A)**, PFS **(B)**, DMFS **(C)** and OS **(D)**. CI, confidence interval; WPRT, whole-pelvic radiotherapy; PORT, prostate-only radiotherapy; BFFS, biochemical failure-free survival; PFS, progression-free survival; DMFS, distant metastasis-free survival; OS, overall survival.

Six studies (9, 10, 26, 29–31) with a total of 3,795 patients examined BFFS at 5 years. The forest plot (**Figure 2A**) indicated that for the patients did not undergo prostatectomy, WPRT was associated with superior BFFS relative to PORT (HR 0.23, CI 0.10–0.52, *p* < 0.001). Similarly, WPRT also improved the 5-year BFFS when the patients had undergone prostatectomy (HR 0.58, CI 0.50–0.68, *p* < 0.001). Overall, WPRT significantly improved 5-year BFFS (HR 0.56, CI 0.48–0.66, *p* < 0.001) compared with PORT (*I* ^2^ = 40%, *p* = 0.14). All patients who did not undergo prostatectomy received ADT, whereas the studies of patients treated with prostatectomy were divided into three subgroups based on the use of ADT. The subgroup analysis of the RP studies showed that WPRT had excellent BFFS than PORT, free from the effect of ADT (**Supplementary Figure 1A**).

### Progression-Free Survival

Progression-free survival (PFS) data were available from four studies (7, 10–12), 3,278 patients. The forest plot (**Figure 2B**) indicated that, in comparison with PORT, WPRT significantly improved PFS for non-RP studies (HR 0.85, CI 0.73–0.98, *p* = 0.03) and RP studies (HR 0.71, CI 0.51–0.98, *p* = 0.04). In the subgroup of non-RP studies, when all patients received ADT, WPRT remained improved PFS (HR 0.83, CI 0.71–0.98, *p* = 0.02). Conversely, when patients received ADT selectively, the results showed no difference in PFS between WPRT and PORT (HR 0.96, CI 0.64–1.43, *p* = 0.84; **Supplementary Figure 1B**).

### Distant Metastasis-Free Survival

Three studies (9, 10, 30) with a total of 3,237 patients reported on DMFS at 5 years. The forest plot (**Figure 2C**) of non-RP studies showed that WPRT was more beneficial for DMFS than PORT (HR 0.35, CI 0.15–0.82, *p* = 0.02). However, for the patients who had undergone RP, no significant difference had been found in DMFS between WPRT and PORT (HR 0.81, CI 0.56–1.17, *p* = 0.26).

### Overall Survival

Three studies (9, 10, 23) (4,099 patients) analyzed OS at 5 years (**Figure 2D**). The forest plot (**Figure 2D**) demonstrated that there was no significant difference in OS between WPRT and PORT, regardless of whether the patient underwent prostatectomy. However, the further subgroup analysis of non-RP studies indicated that when all patients received ADT, WPRT was not superior than PORT in OS (HR 0.93, CI 0.75–1.15, *p* = 0.50). Conversely, when no patients received ADT, WPRT significantly improved OS (HR 0.58, CI 0.38–0.89, *p* = 0.01; **Supplementary Figure 1C**) than PORT.

### Subgroup Analysis of Potential Heterogeneity Factors for Survival Outcomes in Non-RP Studies

Further subgroup analysis of non-RP studies indicated that the PFS and OS of younger patients (age ≤ 66 years) did not benefit from WPRT (*p* = 0.67 and 0.34, respectively; **Table 2**). Meanwhile, long-term ADT did not have significant advantages in improving PFS and OS (*p* = 0.85 and 0.98, respectively) over short-term ADT. Moreover, there was still no significant difference in PFS between the higher-risk (intermediate- and high-risk) and low-risk patients (*p* = 0.34; **Supplementary Figure 2**). Although when pelvic radiation dose was >49 Gy (equivalent-doses-in-2-Gy-fractions, EQD-2), compared with PORT, WPRT significantly improved PFS (HR 0.83, CI 0.70–0.98, *p* = 0.03), and the higher pelvic radiation dose (>49Gy, EQD-2) did not seem to have a significant advantage in improving PFS over dose ≤49 Gy (*p* = 0.53).

**Table 2:** Subgroup analysis of other potential heterogeneity factors for survival outcomes in non-prostatectomy studies.

Heterogeneity factors	Hazard ratio (95% CI) WPRT vs. PORT	*p-*Value	*p*-Value for Interaction	*I* ^2^ (%)
Age > 66 for PFS				
Yes	0.77 [0.60, 1.00]	*p* = 0.05	*p* = 0.67	0
No	0.83 [0.70, 0.98]	*p* = 0.03		
Duration of ADT for PFS				
Long-term ADT	0.88 [0.59, 1.31]	*p* = 0.53	*p* = 0.85	0
Short-term ADT	0.85 [0.72, 0.99]	*p* = 0.04		
Dose >49 Gy* for PFS				
Yes	0.83 [0.70, 0.98]	*p* = 0.03	*p* = 0.53	0
No	0.92 [0.66, 1.02]	*p* = 0.56		
Risk goup for PFS				
Low risk	0.71 [0.41, 1.21]	*p* = 0.21	*p* = 0.34	0
Intermediate and high risk	0.95 [0.75, 1.20]	*p* = 0.66		
Age > 66 for OS				
Yes	0.95 [0.77, 1.18]	*p* = 0.64	*p* = 0.34	0
No	0.55 [0.18, 1.64]	*p* = 0.28		
Duration of ADT for OS				
Long-term ADT	0.92 [0.41, 2.05]	*p* = 0.84	*p* = 0.98	0
Short-term ADT	0.93 [0.75, 1.16]	*p* = 0.52		

WPRT, whole-pelvic radiotherapy; PORT, prostate-only radiotherapy; ADT, androgen deprivation therapy; BT, brachytherapy; BFFS, biochemical failure-free survival; PFS, progression-free survival; DMFS, Distant metastasis-free survival; OS, overall survival.

*Equivalent-doses-in-2-Gy-fractions, EQD-2.

### Gastrointestinal Toxicity

**Figure 3:**
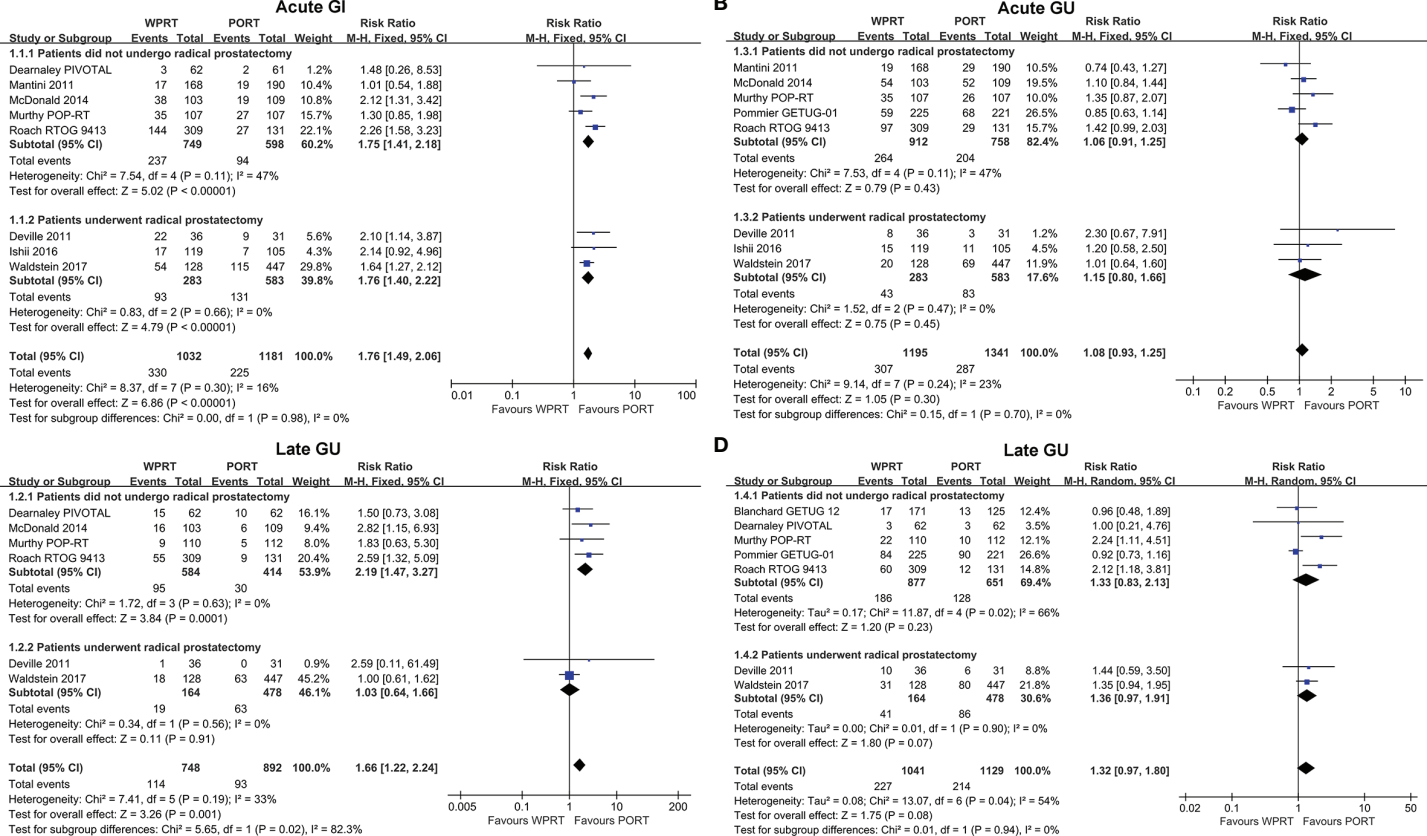
Forest plots of Acute GI **(A)**, Late GI **(B)**, Acute GU **(C)**, and Late GU **(D)**. CI, confidence interval; WPRT, whole-pelvic radiotherapy; PORT, prostate-only radiotherapy; GI, gastrointestinal; GU, genitourinary.

Eight studies (8, 9, 12, 24, 25, 27, 28, 32) evaluated acute GI toxicity and six studies (8, 9, 12, 24, 28, 32) examined late GI toxicity. According to the forest plots (**Figure 3**), WPRT significantly increased the grade 2 or worse (G2+) acute GI toxicity of non-RP studies (RR 1.75, CI 1.41–2.18, *p* < 0.001) and RP studies (RR 1.76, CI 1.40–2.22, *p* < 0.001). The forest plots (**Figure 3B**) illustrated that WPRT also significantly increased G2+ late GI toxicity of non-RP studies (RR 2.19, CI 1.47–3.27, *p* < 0.001).

### Genitourinary Toxicity

Acute GU toxicity was assessed in eight studies (9, 11, 12, 24, 25, 27, 28, 32) and late GU toxicity was evaluated in seven studies (7–9, 11, 12, 24, 32). The forest plots (**Figure 3C**) showed that there was no statistically significant difference in the G2+ acute GU toxicity of non-RP studies (RR 1.06, CI 0.91–1.25, *p* = 0.43) and RP studies (RR 1.15, CI 0.80–1.66, *p* = 0.45). According to the forest plots (**Figure 3D**), there was still no significant difference in the G2+ late GU toxicity of non-RP studies (RR 1.33, CI 0.83–2.13, *p* = 0.23) and RP studies (RR 1.36, CI 0.97–1.91, *p* = 0.07). The risk difference of toxicity has been presented in **Supplementary Figure 3**.

### Subgroup Analysis of Potential Heterogeneity Factors for GI Toxicity and GU Toxicity in Non-RP Studies

**Figure 4:**
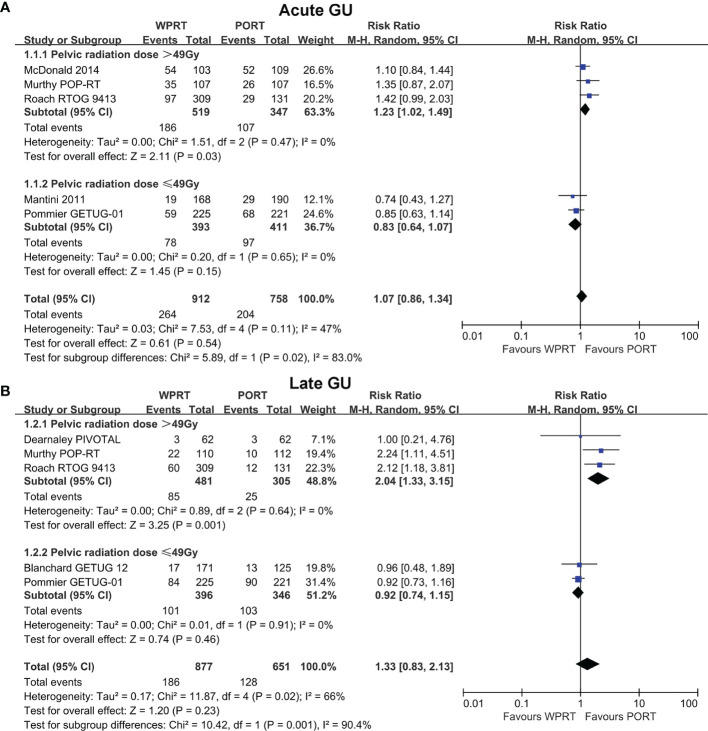
Subgroup analysis of radiation dose for non-RP studies. Acute GU **(A)** and Late GU **(B)** of pelvic radiation dose. CI, confidence interval; WPRT, whole-pelvic radiotherapy; PORT, prostate-only radiotherapy; GU, genitourinary.

Further subgroup analysis of non-RP studies indicated that when pelvic radiation dose was >49 Gy (EQD-2), WPRT significantly increased the G2+ acute GU toxicity (RR 1.26, CI 1.04–1.54, *p* = 0.02; **Figure 4A**) and late GU toxicity (RR 2.04, CI 1.33–3.15, *p* = 0.001; **Figure 4B**). On the contrary, when pelvic radiation dose was ≤49 Gy (EQD-2), there was no significant difference in the G2+ acute GU toxicity (RR 0.82, CI 0.63–1.06, *p* = 0.14; **Figure 4A**) and late GU toxicity (RR 0.92, CI 0.74-1.15, *p* = 0.46; **Figure 4B**). Moreover, the higher pelvic radiation dose (>49 Gy, EQD-2) had a significant increase in G2+ acute and late GU toxicity of WPRT (*p* = 0.009 and 0.001, respectively; **Figure 4**). On the other hand, meta-regression analysis showed no significant correlation between prostate radiation dose and GU toxicity (**Supplementary Table 2**).

**Figure 5:**
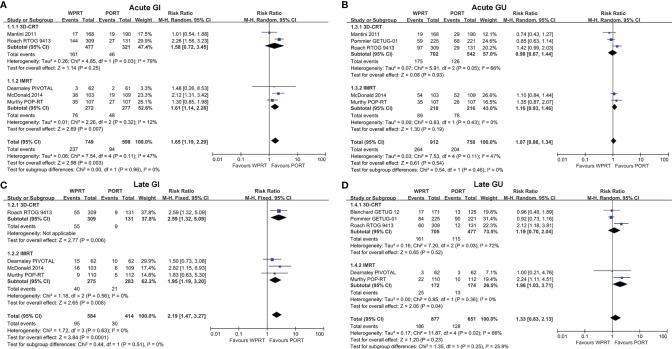
Subgroup analysis of radiotherapy technology for non-RP studies. Acute GI **(A)**, Late GI **(B)**, Acute GU **(C)**, and Late GU **(D)**. CI, confidence interval; WPRT, whole-pelvic radiotherapy; PORT, prostate-only radiotherapy; GI, gastrointestinal; GU, genitourinary.

In addition, subgroup analysis of radiotherapy technology indicated that compared with intensity-modulated radiation therapy (IMRT), three-dimensional conformal radiotherapy (3D-CRT) increased G2+ late GI toxicity of WPRT without significant difference (**Figure 5**). The risk difference of radiation dose and radiotherapy technology on GU toxicity has been presented in **Supplementary Figures 4, 5**.

### Quality Assessment and Risk of Bias

We used the Cochrane Risk of Bias Tool to evaluate the risk of bias in RCTs, and the results showed that most of the evidence was moderate-to-good quality (**Supplementary Figure 6**). The included CRS demonstrated Newcastle-Ottawa scores consistent with a low to moderate risk of bias (**Supplementary Table 3**). We assessed the publication bias using funnel plots comparing effect size and measure of precision across our primary analysis (**Supplementary Figure 7**). Although some comparisons involved a small number of studies, we did not identify evidence of a publication bias.

## Discussion

There has been considerable controversy over whether to choose whole pelvic radiotherapy (WPRT) or prostate-only radiotherapy (PORT) for localized prostate cancer (37). This meta-analysis included six randomized controlled studies (RCTs) (7–12) and ten cohort studies (CRS) (23–32). Of the 16 studies, 9 studies did not perform radical prostatectomy (non-RP), and the patients of the other 7 studies received radical prostatectomy (RP). The effects of WPRT and PORT on biochemical failure-free survival (BFFS), progression-free survival (PFS), distant metastasis-free survival (DMFS), and overall survival (OS) were analyzed. The BFFS is strictly based on serum prostate-specific antigen (PSA) level and is a crucial outcome indicator for evaluating prostate cancer (38). Some adverse survival events of localized prostate cancer were caused by local progression, regional or nodal failure, and distant metastasis. Therefore, many studies also supplemented PFS as an outcome indicator (7, 10–12, 39). Recent studies found that DMFS was a strong surrogate of overall survival in localized prostate cancer that was associated with a significant risk of death from prostate cancer (40, 41). This meta-analysis revealed that compared with PORT, WPRT significantly improved 5-year BFFS and PFS, and it was irrelevant to whether the patients had undergone radical prostatectomy (RP). In addition, for the patients who did not receive RP, the 5-year DMFS of WPRT was better than that of PORT.

ADT and radical prostatectomy (RP) were factors that improved the survival rates of prostate cancer when comparing WPRT and PORT. Radiotherapy (RT) combined with ADT is the recommended radical treatment for high-risk localized prostate cancer (42–44). The Southwest Oncology Group (SWOG) 8794 (45) indicated that RT combined with RP was more beneficial to BFFS and DMFS. In addition, radiation dose, radiotherapy technology, and the extent of radiotherapy pelvic lymph node coverage also have an impact on survival and toxicity (37, 46). This meta-analysis attempted to evaluate the effect of WPRT on the survival and toxicity of localized prostate cancer compared with PORT under the influence of these factors.

The National Comprehensive Cancer Network (NCCN) recommends that if the prostate tumor is aggressive, ADT should be routinely used. However, RTOG 9601 cleared that with the use of ADT, side effects also occurred (such as gynecomastia) (47). Ramey et al. (30) revealed a potentially additive effect to WPRT and ADT. WPRT+ADT was significantly beneficial for BFFS (HR = 0.56) compared with PORT+ADT, and the addition of ADT to WPRT could further improve BFFS than WPRT alone. In the postoperative setting, although there is no level 1 evidence for choosing WPRT or PORT, more than 70% of radiation oncologists suggest that WPRT should be used after prostatectomy (30, 48). The subgroup analysis of this study indicated that whether combined with ADT or not, WPRT significantly improved BFFS of patients undergoing RP compared to PORT (**Supplementary Figure 1A**).

Although a host’s immune system may be able to remove a single tumor cell, it may be reasonable to advocate inclusion of the WPRT to eradicate or diminish residual cells with metastatic potential (49). However, it remains unclear how much of the survival outcomes that may be improved with the addition of WPRT is caused by the effect on micrometastasis or secondarily results from the improved local tumor control (50). On the other hand, Conventional imaging modalities perform poorly in detecting lymph node metastases from prostate cancer, and 25%–40% of patients who undergo radical prostatectomy with an extended pelvic lymphadenectomy have these identified by histology (51). Since patients with lymph node metastases have been shown to benefit from pelvic radiotherapy, these false-negative diagnosis errors may also be one of the reasons that WPRT has an advantage over PORT in the clinical benefit. We further analyzed the possible dependence between the clinical benefit of WPRT and patient characteristics (including age, Gleason score, and Nodal Risk). The results showed that younger patients (age ≤66 years) seemed to derive a greater benefit for BFFS and DMFS with WPRT (*p* = 0.03 and 0.01, respectively). However, the Gleason score and nodal risk of localized prostate cancer did not have a significant effect on the clinical benefit of WPRT (**Table 3**).

**Table 3:** The possible dependence between the clinical benefit of WPRT and patient characteristics in RCTs.

Heterogeneity factors	RCT	No. of events/Total no.WPRT PORT		Hazard ratio (95% CI) WPRT vs. PORT	*p*-Value
Age years for BFFS	POP-RT				
≤66		2/59	22/58	0.08 [0.02, 0.35]	0.03
>66		5/51	7/54	0.66 [0.21, 2.10]	
Gleason for BFFS	POP-RT				
<8		2/57	9/56	0.22 [0.05, 1.01]	0.88
≥8		5/53	20/56	0.24 [0.09, 0.64]	
Nodal Risk for BFFS	POP-RT				
≤40%		4/59	11/60	0.36 [0.12, 1.14]	0.28
<40%		3/51	18/52	0.15 [0.04, 0.50]	
Gleason for PFS	GETUG 12				
<8		52/111	27/89	1.26 [0.76, 2.09]	0.20
≥8		53/97	30/61	0.95 [0.59, 1.53]	
Age years for DMFS	POP-RT				
≤66		2/59	17/58	0.11[0.03, 0.49]	0.01
>66		5/51	3/54	1.63[0.39, 6.85]	
Gleason for DMFS	POP-RT				
<8		2/57	6/56	0.32[0.06, 1.60]	0.88
≥8		5/53	14/56	0.37[0.13, 1.04]	

WPRT, whole-pelvic radiotherapy; PORT, prostate-only radiotherapy; RCT, randomized controlled trial; BFFS, biochemical failure-free survival; PFS, progression-free survival; DMFS, distant metastasis-free survival.

The extent and adequacy of radiotherapy pelvic lymph node coverage may also be a critical factor affecting the benefit of WPRT in prostate cancer (37). Proponents of WPRT argue that the lack of benefit demonstrated by the GETUG-01 and RTOG 9413 trials may be due in part to inadequate coverage of the pelvic lymph nodes, given that the respective superior field borders of S1/2 and L5/S1 would not provide full dose coverage to the entire superior pelvic lymph node basins (52). Spratt et al. (53) looked at lymph node recurrence patterns after external beam radiotherapy of the prostate in men who did not have their lymph nodes treated. It was found that there was a high incidence of pelvic lymph node recurrences above the internal and external iliac lymph node regions. The recent Phase III trial, POP-RT, evaluated the benefit of WPRT with extended superior coverage to L4/5, and pointed out that WPRT improved the BFFS, disease-free survival (DFS), and DMFS compared with PORT, but resulted in a significant increase in late grade 2 or worse (G2+) genitourinary (GU) toxicity (**Table 4**).

**Table 4:** Pelvic CTV lymph node volume and outcomes of Non-RP studies.

First Author	Institution	Area of Pelvic CTV Lymph Node	Key Findings
Blanchard	GETUG	The upper limit of the pelvis could be either S1-S2 (small pelvis) or L5-S1 (large pelvis)	There was no association between biochemical PFS and the use of WPRT
Braunstein	Harvard	Beginning at the bifurcation of the aorta to the common iliac arteries (approximating vertebral levels L4 and L5) and included internal and external iliac chains	A decreased risk of ACM was noted with the use of WPRT versus PORT. However, a combination of WPRT and ADT did not further improve ACM compared with PORT with ADT
Dearnaley	CRUK	Lower border L5 on sagittal CT	WPRT had a modest side effect profile.
Ishii	Tane General Hospital, Japan	Obturator vessels, the common, external and internal iliac vessels	WPRT resulted in no significant increase in acute GU toxicity when compared with PORT
Mantini	Catholic University, Italy	Presacral, obturator, internal iliac, and external iliac chains	No significant differences were seen in acute and late GI and GU toxicity among the patients treated with WPRT or PORT
McDonald	University of Alabama, USA	Starting at L5-S1 junction	WPRT increases the rates of acute and late GI toxicity
Murthy	Tata Memorial Centre, India	Starting at L4-5 junction to include bilateral common iliac, external iliac, internal iliac, presacral	WPRT improved BFFS and DFS as compared with PORT, but OS did not appear to differ. WPRT resulted in increased G2+ late GU toxicity as compared to PORT
Pommier	GETUG	Routine radiation field coverage to the S1/2 interspace	WPRT was well tolerated but did not improve PFS.
Roach	RTOG	The pelvic CTV lymph node volumes at the L5/S1 interspace (the level of the distal common iliac and proximal presacral lymph nodes)	NHT plus WPRT improved PFS compared with NHT plus PORT albeit increased risk of grade 3 or worse intestinal toxicity

GETUG, French Genitourinary Study Group; CRUK, Cancer Research UK; RTOG, Radiation Therapy Oncology Group; WPRT, whole-pelvic radiotherapy; PORT, prostate-only radiotherapy; PFS, progression-free survival; ACM, all-cause mortality; ADT, androgen deprivation therapy; NR, not reported; GU, genitourinary; GI, gastrointestinal; BFFS, biochemical failure-free survival; DFS, disease-free survival; OS, overall survival; G2+, grade 2 or worse; NHT, neoadjuvant hormonal therapy.

The RTOG 9413 (12) reported that WPRT plus neoadjuvant hormonal therapy (NHT) significantly improved PFS and BFFS and there was no difference between groups in grade 3 or worse (G3+) late GU toxicity, but caused a significant increase in the risk of G3+ gastrointestinal (GI) toxicity. Our meta-analysis further demonstrated that the toxicity of WPRT on G2+ acute and late GU was related to whether the pelvic radiation dose was >49 Gy (EQD-2). On the other hand, the toxicities of GI and GU are also related to radiotherapy technology (54). Wortel et al. concluded that intensity-modulated radiation therapy (IMRT) resulted in significant reductions in G2+ acute and late GI toxicity and acute GU toxicity compared to three-dimensional conformal radiation therapy (3D-CRT) (55, 56). Our subgroup analysis of radiotherapy technology indicated that compared with IMRT, 3D-CRT increased G2+ late GI toxicity of WPRT without significant difference (**Figure 5**).

This study has several limitations. First, subgroup analyses were restricted by the study-level nature of the data. Second, a follow-up only longer than 5 years is inadequate to thoroughly evaluate the impact of one therapeutic approach over another, especially with respect to “harder clinical end-points” such as DMFS, overall, and cancer-specific survival. At present, two randomized controlled trials of longer than 10 years of follow-up have been published. The GETUG-01 showed that pelvic nodes irradiation did not statistically improve 10-year event-free survival (EFS) or OS in the whole population but may be beneficial in selected low- and intermediate-risk prostate cancer patients treated with exclusive radiation therapy (11). The RTOG 9413 demonstrated that WPRT plus NHT improved 10-year PFS compared with PORT plus NHT (12). More research publications with longer than 10 years of follow-up are needed for the next longer follow-up meta-analysis.

In conclusion, this meta-analysis demonstrates that WPRT significantly improved 5-year BFFS and PFS compared with PORT in localized prostate cancer. Moreover, for the patients who did not receive RP, the 5-year DMFS of WPRT was better than that of PORT. However, WPRT significantly increased not only the grade 2 or worse (G2+) acute GI toxicity of non-RP studies and RP studies, but also the G2+ late GI toxicity of non-RP studies. Subgroup analysis of non-RP studies found that when the pelvic radiation dose was >49 Gy (equivalent-doses-in-2-Gy-fractions, EQD-2), WPRT was more beneficial to PFS than PORT, but significantly increased the risk of G2+ acute and late GU toxicity.

## Data Availability Statement

The original contributions presented in the study are included in the article/**Supplementary Material**. Further inquiries can be directed to the corresponding authors.

## Author Contributions

Study concept and design: FJ and SW. Data collection and collation: SW and WT. Statistical analysis: SW, WT, and HL. Writing—original draft: SW. Writing—review and editing: all authors. Study supervision: YW, FJ, and HL. All authors contributed to data analysis, drafting or revision of this paper, and approved the final version. The corresponding authors prove that all listed authors meet the authorship criteria, and that no other eligible authors have been omitted.

## Funding

This work was supported by the National Natural Science Foundation of China (Nos. 81972857, 11805025 and 11575038).

## Conflict of Interest

The authors declare that the research was conducted in the absence of any commercial or financial relationships that could be construed as a potential conflict of interest.

## Publisher’s Note

All claims expressed in this article are solely those of the authors and do not necessarily represent those of their affiliated organizations, or those of the publisher, the editors and the reviewers. Any product that may be evaluated in this article, or claim that may be made by its manufacturer, is not guaranteed or endorsed by the publisher.
