# Supplementary material for: Efficacy and Toxicity of Whole Pelvic Radiotherapy Versus Prostate-Only Radiotherapy in Localized Prostate Cancer: A Systematic Review and Meta-Analysis
Source: Front Oncol. 2022 Jan 27;11:796907. doi: 10.3389/fonc.2021.796907 (PMC8828576; doi:10.3389/fonc.2021.796907)
Supplement: Supplementary file 1 [file DataSheet_1.docx]

**Supplementary Table 1. Number of citations by each database and trial register searched**

| **Databases and Trial registers** | **Citations** |
| --- | --- |
| **Databases：** |  |
| PubMed | 813 |
| Cochrane | 391 |
| Embase | 2474 |
| MEDLINE | 1002 |
| **Total (databases)** | 4680 |
|  |  |
| **Trial registers:** |  |
| USA (ClinicalTrials.gov) | 52 |

**Search Strategy of Cochrane, PubMed, Embase, Medline databases and ClinicalTrials.gov**

**PubMed**

((((("Prostatic Neoplasms"[Mesh]) OR (((((((((((((((((Prostate Neoplasms[Title/Abstract]) OR (Neoplasms, Prostate[Title/Abstract])) OR (Neoplasm, Prostate[Title/Abstract])) OR (Prostate Neoplasm[Title/Abstract])) OR (Neoplasms, Prostatic[Title/Abstract])) OR (Neoplasm, Prostatic[Title/Abstract])) OR (Prostatic Neoplasm[Title/Abstract])) OR (Prostate Cancer[Title/Abstract])) OR (Cancer, Prostate[Title/Abstract])) OR (Cancers, Prostate[Title/Abstract])) OR (Prostate Cancers[Title/Abstract])) OR (Cancer of the Prostate[Title/Abstract])) OR (Prostatic Cancer[Title/Abstract])) OR (Cancer, Prostatic[Title/Abstract])) OR (Cancers, Prostatic[Title/Abstract])) OR (Prostatic Cancers[Title/Abstract])) OR (Cancer of Prostate[Title/Abstract])))) AND (("Radiotherapy"[Mesh]) OR ((((((((((((((((((Radiotherapies[Title/Abstract]) OR (Radiation Therapy[Title/Abstract])) OR (Radiation Therapies[Title/Abstract])) OR (Therapies, Radiation[Title/Abstract])) OR (Therapy, Radiation[Title/Abstract])) OR (Radiation Treatment[Title/Abstract])) OR (Radiation Treatments[Title/Abstract])) OR (Treatment, Radiation[Title/Abstract])) OR (Radiotherapy, Targeted[Title/Abstract])) OR (Radiotherapies, Targeted[Title/Abstract])) OR (Targeted Radiotherapies[Title/Abstract])) OR (Targeted Radiotherapy[Title/Abstract])) OR (Targeted Radiation Therapy[Title/Abstract])) OR (Radiation Therapies, Targeted[Title/Abstract])) OR (Targeted Radiation Therapies[Title/Abstract])) OR (Therapies, Targeted Radiation[Title/Abstract])) OR (Therapy, Targeted Radiation[Title/Abstract])) OR (Radiation Therapy, Targeted[Title/Abstract]))))) AND ((((("Pelvis"[Mesh]) OR ((Pelvic[Title/Abstract]) OR (Pelvic Region[Title/Abstract]) OR (Region, Pelvic[Title/Abstract]))) OR (WPRT[Title/Abstract])) OR (Whole-Pelvic[Title/Abstract]))))) AND (((((Efficacy[Title/Abstract]) OR (Safety[Title/Abstract])) OR (Survival[Title/Abstract])) OR (Toxicity[Title/Abstract])) OR (Toxicities[Title/Abstract]))

**Cochrane**

#1 MeSH descriptor: [Prostatic Neoplasms] explode all trees

#2 (Prostate Neoplasms):ti,ab,kw OR (Neoplasms, Prostate):ti,ab,kw OR (Neoplasm, Prostate):ti,ab,kw OR (Prostate Neoplasm):ti,ab,kw OR (Prostate Cancer):ti,ab,kw (Word variations have been searched)

#3 #1 OR #2

#4 MeSH descriptor: [Radiotherapy] explode all trees

#5 (Radiotherapies):ti,ab,kw OR (Radiation Therapy):ti,ab,kw OR (Radiation Therapies):ti,ab,kw OR (Therapies, Radiation):ti,ab,kw OR (Radiation Treatment):ti,ab,kw (Word variations have been searched)

#6 #4 OR #5

#7 MeSH descriptor: [Pelvis] explode all trees

#8 (Pelvic Region):ti,ab,kw OR (Region, Pelvic):ti,ab,kw OR (Pelvic):ti,ab,kw OR (WPRT):ti,ab,kw OR (Whole-Pelvic):ti,ab,kw (Word variations have been searched)

#9 #7 OR #8

#10 (Efficacy):ti,ab,kw OR (Safety):ti,ab,kw OR (survival):ti,ab,kw OR (Toxicity):ti,ab,kw OR (Toxicities):ti,ab,kw (Word variations have been searched)

#11 #3 AND #6 AND #9 AND # 10

**Embase**

#1 'radiotherapy'/exp OR radiotherapies:ab,ti OR 'radiation therapy':ab,ti OR 'radiation therapies':ab,ti OR 'therapies, radiation':ab,ti OR 'therapy, radiation':ab,ti OR 'radiation treatment':ab,ti OR 'radiation treatments':ab,ti OR 'treatment, radiation':ab,ti OR 'radiotherapy, targeted':ab,ti OR 'radiotherapies, targeted':ab,ti OR 'targeted radiotherapies':ab,ti OR 'targeted radiotherapy':ab,ti OR 'targeted radiation therapy':ab,ti OR 'radiation therapies, targeted':ab,ti OR 'targeted radiation therapies':ab,ti OR 'therapies, targeted radiation':ab,ti OR 'therapy, targeted radiation':ab,ti OR 'radiation therapy, targeted':ab,ti

#2 'prostate neoplasms'/exp OR 'prostate cancer'/exp OR 'prostate neoplasms':ab,ti OR 'neoplasms, prostate':ab,ti OR 'neoplasm, prostate':ab,ti OR 'prostate neoplasm':ab,ti OR 'neoplasms, prostatic':ab,ti OR 'neoplasm, prostatic':ab,ti OR 'prostatic neoplasm':ab,ti OR 'prostate cancer':ab,ti OR 'cancer, prostate':ab,ti OR 'cancers, prostate':ab,ti OR 'prostate cancers':ab,ti OR 'cancer of the prostate':ab,ti OR 'prostatic cancer':ab,ti OR 'cancer, prostatic':ab,ti OR 'cancers, prostatic':ab,ti OR 'prostatic cancers':ab,ti OR 'cancer of prostate':ab,ti

#3 'pelvis'/exp OR 'pelvic region':ab,ti OR 'region, pelvic':ab,ti OR pelvic:ab,ti OR wprt:ab,ti OR 'whole pelvic':ab,ti

#4 'safety'/exp OR 'toxicity'/exp OR 'survival'/exp OR efficacy:ab,ti OR survival:ab,ti OR toxicity:ab,ti OR toxicities:ab,ti

#5 #1#2#3#4

**MEDLINE**

#1 ((((((((((((((((MeSH HEADING:exp: (Prostatic Neoplasms) OR TOPIC: (Prostate Neoplasms) ) OR TOPIC: (Neoplasms, Prostate) ) OR TOPIC: (Neoplasm, Prostate) ) OR TOPIC: (Prostate Neoplasm) ) OR TOPIC: (Neoplasms, Prostatic) ) OR TOPIC: (Neoplasm, Prostatic) ) OR TOPIC: (Prostatic Neoplasm) ) OR TOPIC: (Prostate Cancer) ) OR TOPIC: (Cancer, Prostate) ) OR TOPIC: (Cancers, Prostate) ) OR TOPIC: (Prostate Cancers) ) OR TOPIC: (Cancer of the Prostate) ) OR TOPIC: (Prostatic Cancer) ) OR TOPIC: (Cancer, Prostatic) ) OR TOPIC: (Cancers, Prostatic) ) OR TOPIC: (Prostatic Cancers) ) OR TOPIC: (Cancer of Prostate)

#2 (((((((((((((((((MeSH HEADING:exp: (Radiotherapy) OR TOPIC: (Radiotherapies) ) OR TOPIC: (Radiation Therapy) ) OR TOPIC: (Radiation Therapies) ) OR TOPIC: (Therapies, Radiation) ) OR TOPIC: (Therapy, Radiation) ) OR TOPIC: (Radiation Treatment) ) OR TOPIC: (Radiation Treatments) ) OR TOPIC: (Treatment, Radiation) ) OR TOPIC: (Radiotherapy, Targeted) ) OR TOPIC: (Radiotherapies, Targeted) ) OR TOPIC: (Targeted Radiotherapies) ) OR TOPIC: (Targeted Radiotherapy) ) OR TOPIC: (Targeted Radiation Therapy) ) OR TOPIC: (Radiation Therapies, Targeted) ) OR TOPIC: (Targeted Radiation Therapies) ) OR TOPIC: (Therapies, Targeted Radiation) ) OR TOPIC: (Therapy, Targeted Radiation) ) OR TOPIC: (Radiation Therapy, Targeted)

#3 ((((MeSH HEADING:exp: (Pelvis) OR TOPIC: (Pelvic Region) ) OR TOPIC: (Region, Pelvic) ) OR TOPIC: (Pelvic) ) OR TOPIC: (WPRT) ) OR TOPIC: (Whole-Pelvic)

#4((((TOPIC: (Efficacy) OR TOPIC: (Safety) ) OR TOPIC: (Survival) ) OR TOPIC: (Toxicity) ) OR TOPIC: (Toxicities) )

#5 #1#2#3#4

**clinicaltrials.gov/**

('prostate cancer'/exp OR 'prostate cancer') AND ('radiotherapy'/exp OR radiotherapy) AND ('whole pelvic' OR (whole AND ('pelvic'/exp OR pelvic)))

52 trial

**Supplementary Figure 1. Subgroup analysis of the use of ADT. BFFS (A) of RP studies, PFS (B) and OS (C) of non-RP studies.** CI=confidence interval; WPRT=whole-pelvic radiotherapy; PORT=prostate-only radiotherapy; RP=radical prostatectomy; BFFS=biochemical failure-free survival; PFS=progression-free survival; OS=overall survival.


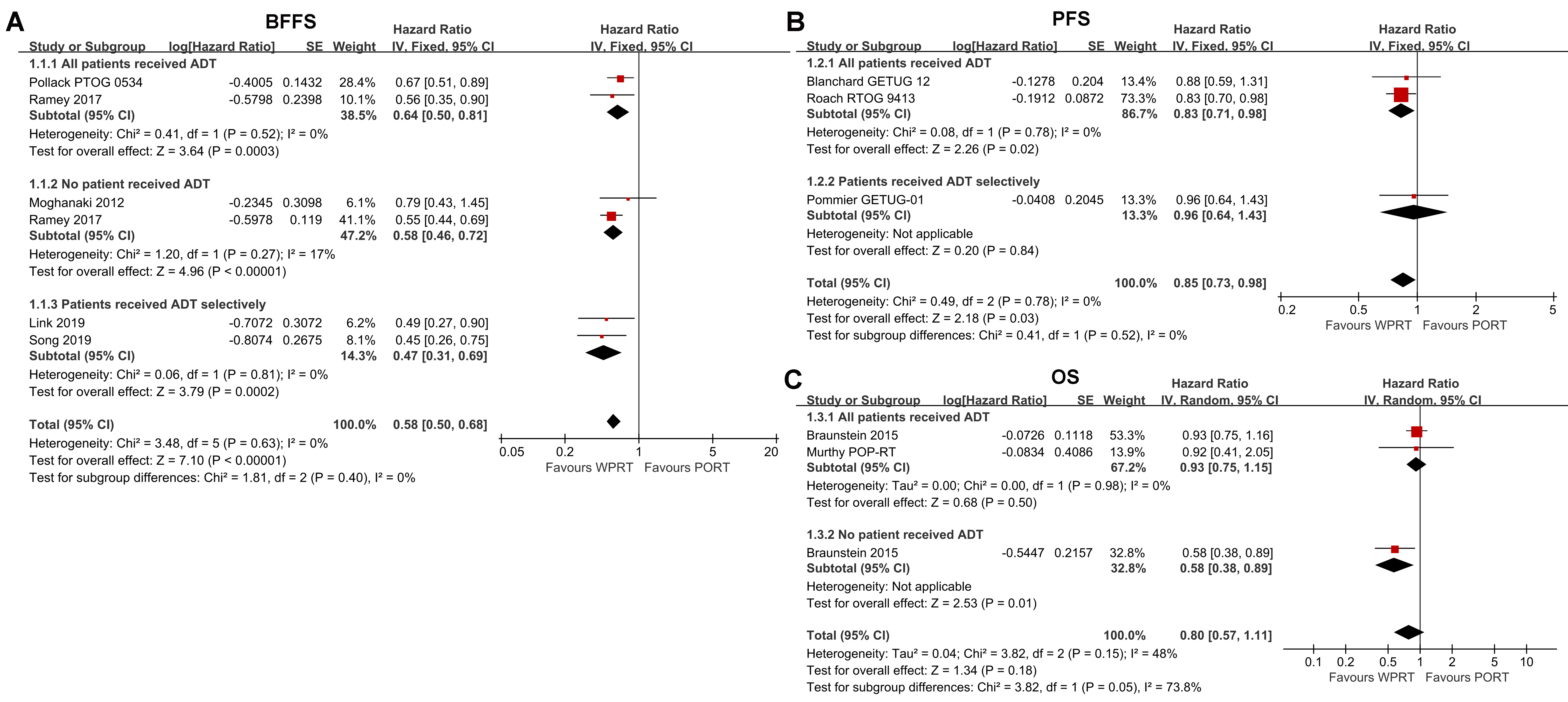


**Supplementary Figure 2. Subgroup analysis of the risk group in PFS.** CI=confidence interval; WPRT=whole-pelvic radiotherapy; PORT=prostate-only radiotherapy; PFS=progression-free survival.

**
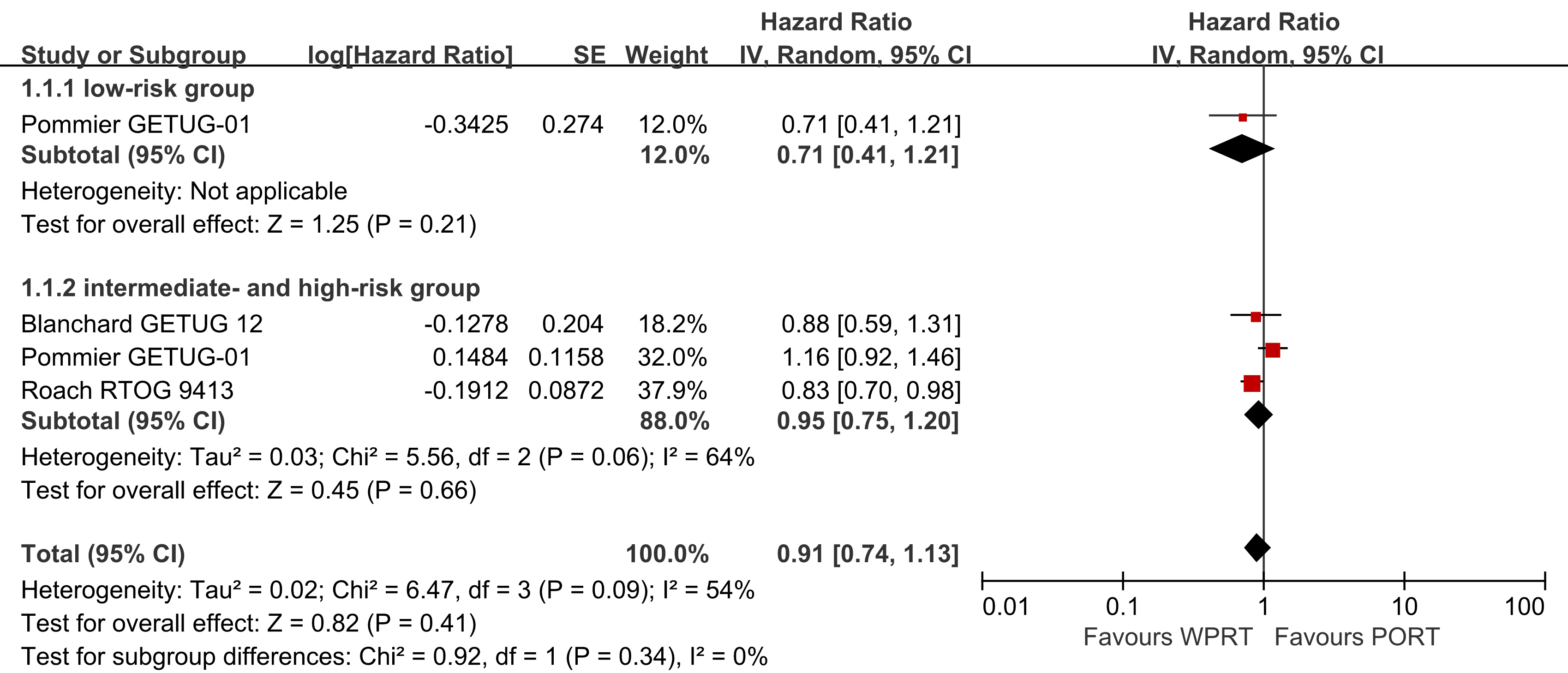
**

**Supplementary Figure 3. The risk difference of toxicity.** **Acute GI (A), Late GI (B), Acute GU (C) and Late GU (D).** CI=confidence interval; WPRT=whole-pelvic radiotherapy; PORT=prostate-only radiotherapy; GI=gastrointestinal; GU=genitourinary.


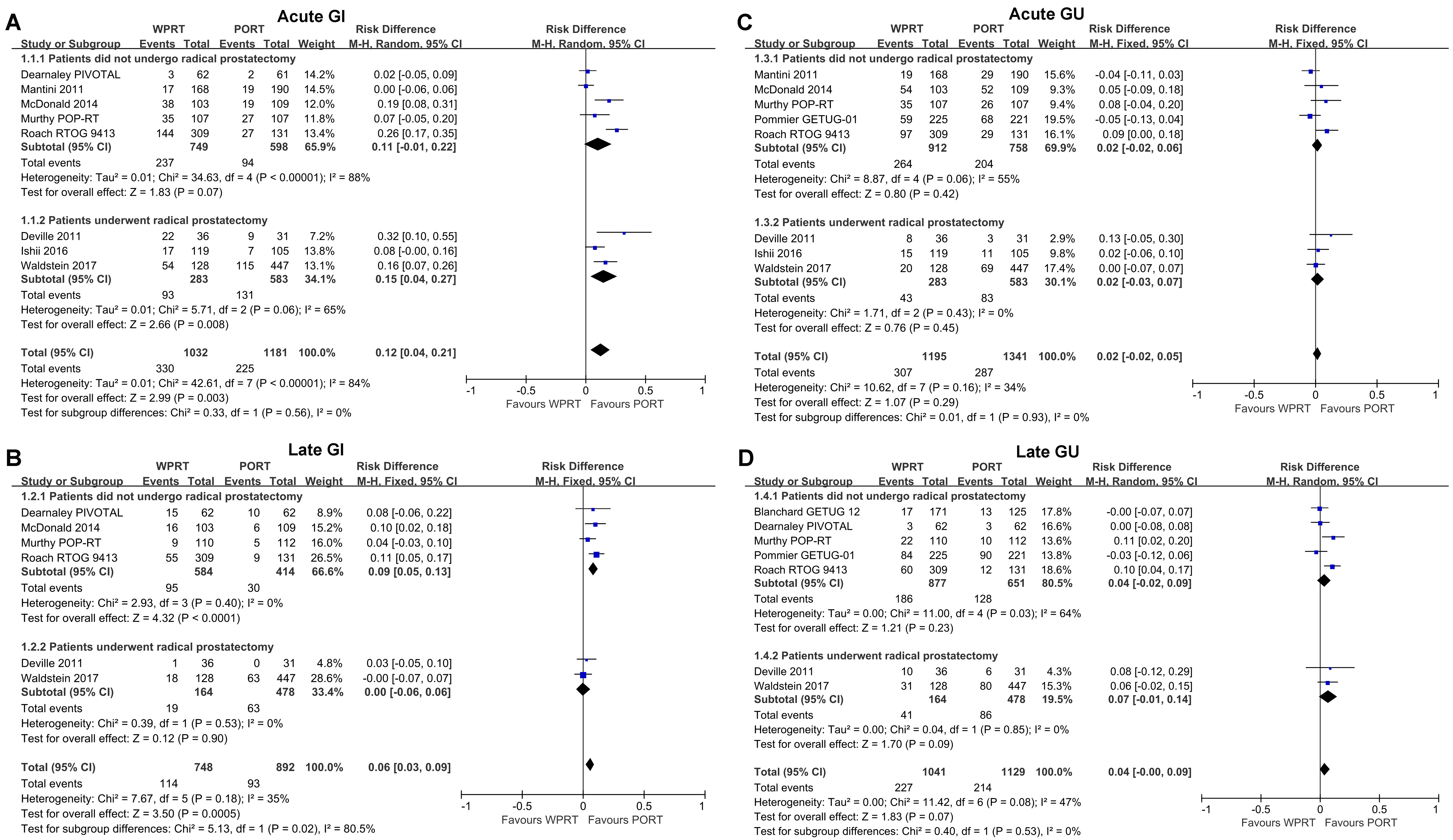


**Supplementary Table 2.** Meta-regression analysis of the correlation between prostate radiation dose and GU toxicity.

| Log ES | Coef. | Std. Err. | t | P>\|t\| | [95% Conf. | Interval] |
| --- | --- | --- | --- | --- | --- | --- |
| Prostate radiation dose | 0.001 | 0.046 | 0.03 | 0.976 | -0.116 | 0.119 |
| _cons | -0.046 | 3.251 | -0.01 | 0.989 | -8.403 | 8.311 |

Meta-regression Number of obs=7

REML estimate of between-study variance tau2=0.034

% residual variation due to heterogeneity I-squared res=33.19%

Proportion of between-study variance explained Adj R-squared=-67.89%

**Supplementary Figure 4. The risk difference of radiation dose for non-RP studies.** **Acute GU (A) Late GU (B) of pelvic radiation dose, Acute GU (C) and Late GU (D) of prostate radiation dose.** CI=confidence interval; WPRT=whole-pelvic radiotherapy; PORT=prostate-only radiotherapy; GU=genitourinary.


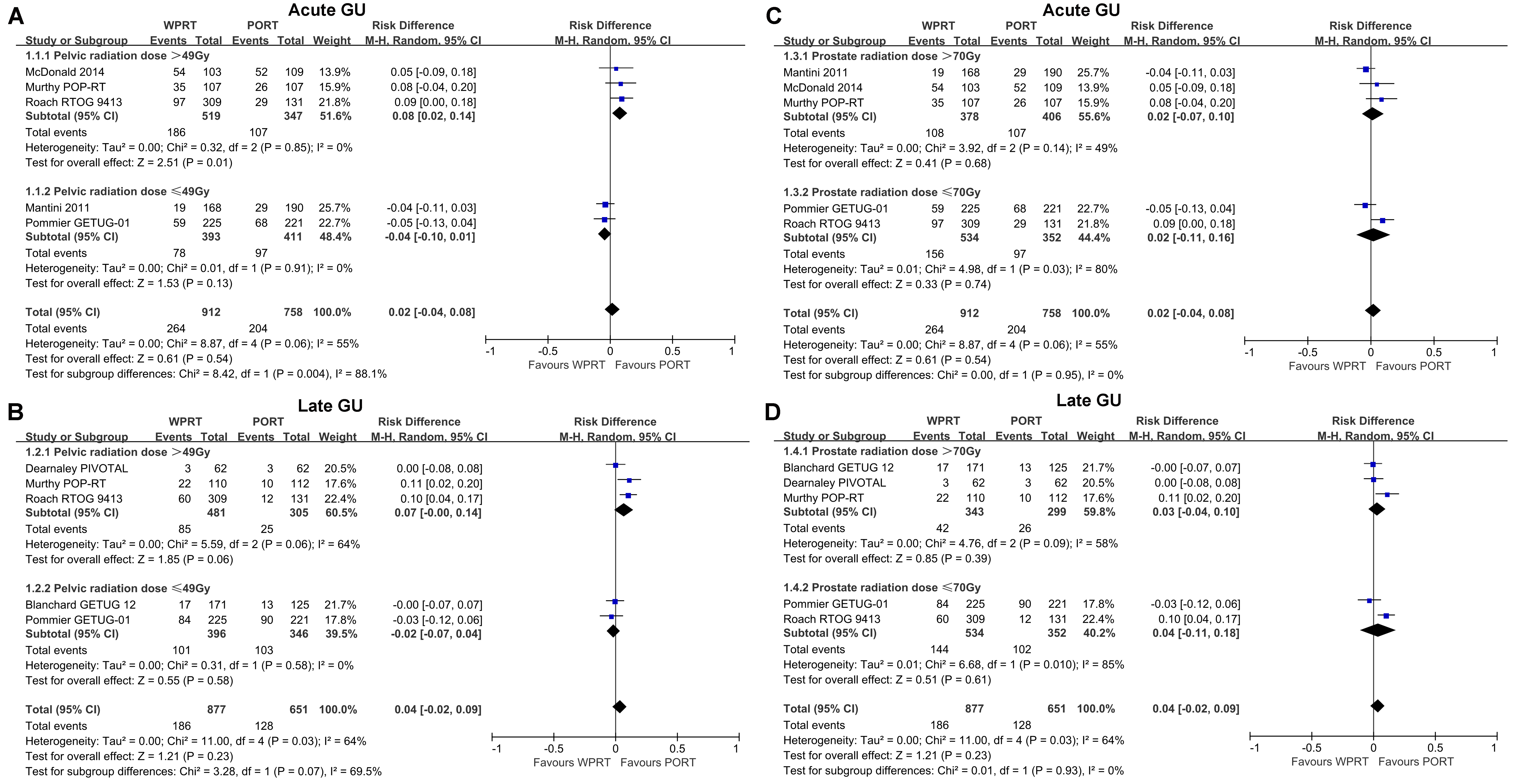


**Supplementary Figure 5. The risk difference of radiotherapy technology for non-RP studies.** **Acute GI (A), Late GI (B), Acute GU (C) and Late GU (D).** CI=confidence interval; WPRT=whole-pelvic radiotherapy; PORT=prostate-only radiotherapy; GI=gastrointestinal; GU=genitourinary.


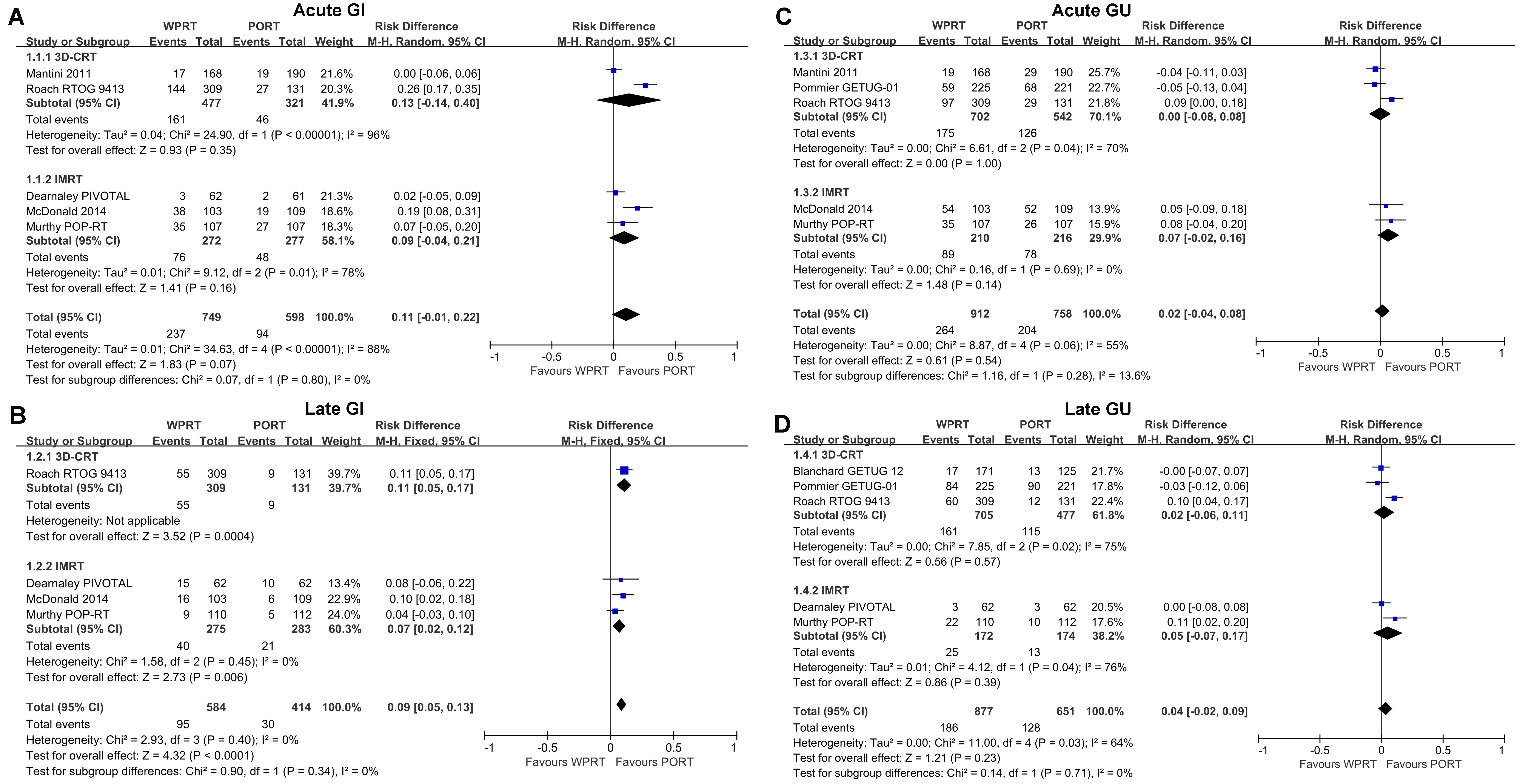


**Supplementary Figure 6. Cochrane Handbook for Systematic Reviews of Interventions of the RCTs.** Risk of bias graph: review authors’ judgments about each risk of bias item presented as percentages across all included studies (A); Risk of bias summary: review authors’ judgments about each risk of bias item for each included study (B).


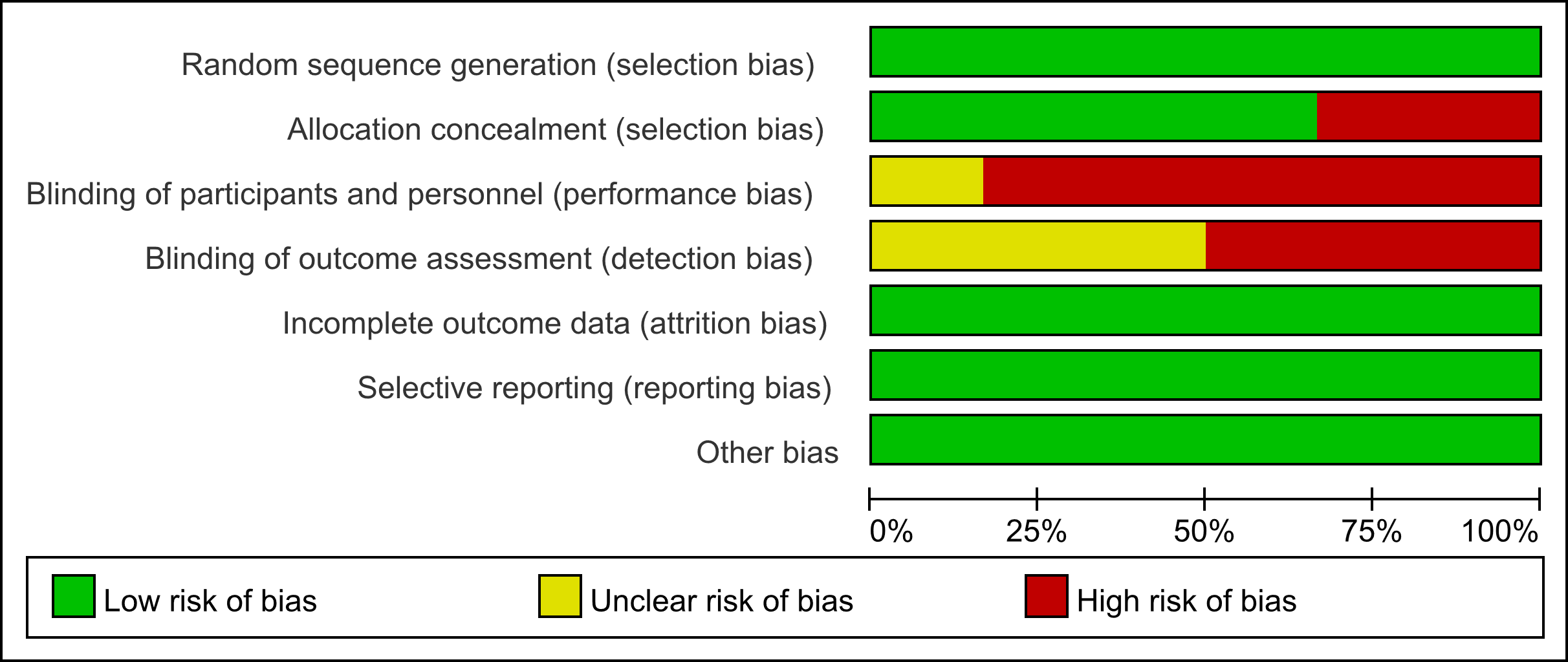


**Fig**. **A** **Risk of Bias Graphs:** Review Authors’ Judgments About Each Risk of Bias Item Presented as Percentages Across All 2 3 Randomized Trials


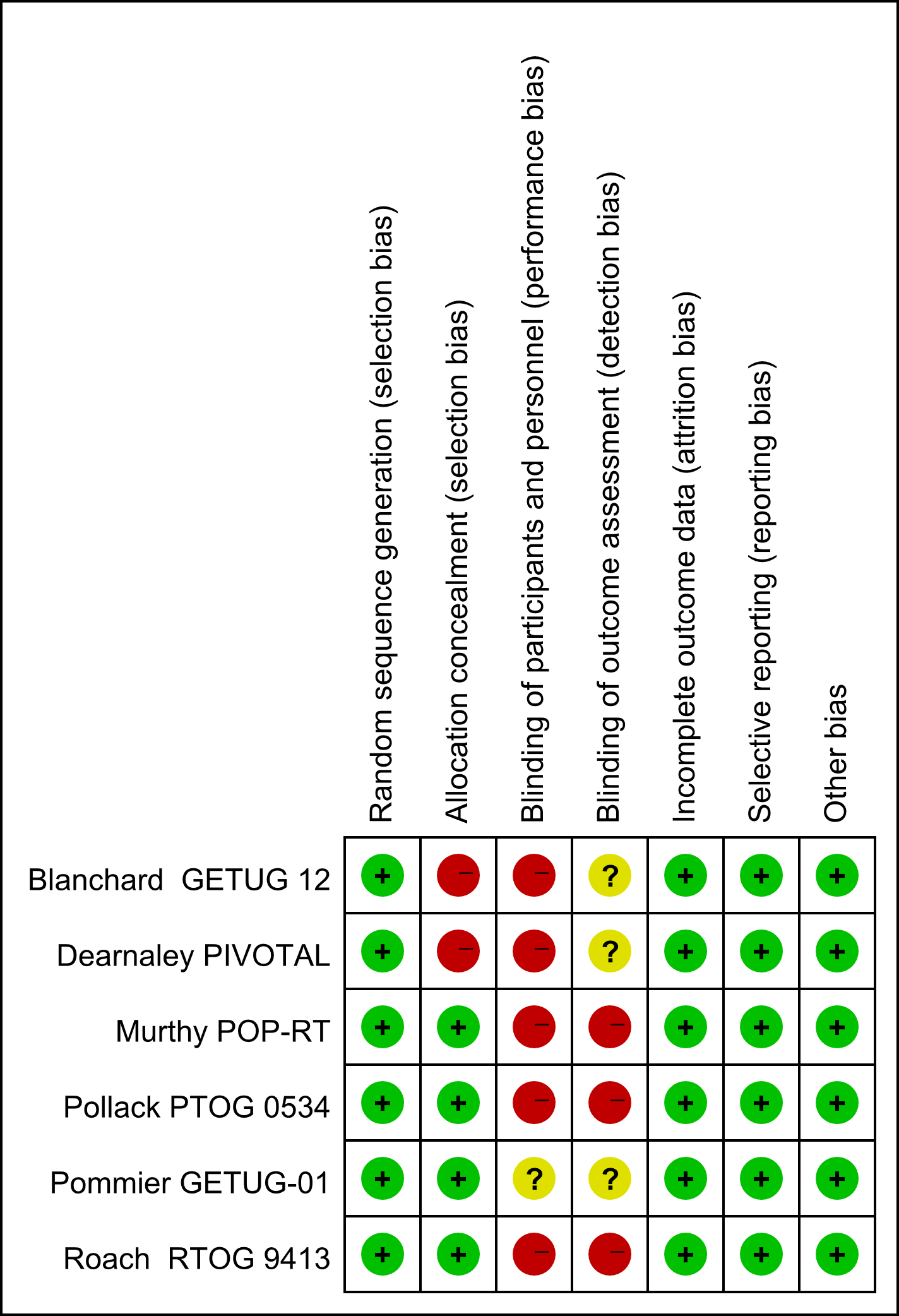


**Fig**. **B** **Risk of Bias Summary:** Review Authors’ Judgments About Each Risk of Bias Item for Each Included Study

**Supplementary Table 3. Newcastle-Ottawa Scale for risk of bias assessment of the CRS**

| Study | Selection | | | | Compar-ability | Outcome | | | Overall |
| --- | --- | --- | --- | --- | --- | --- | --- | --- | --- |
|  | Representativeness  of exposed Cohort | Selection of  nonexposed | Ascertainment  of exposure | Outcome not present at start |  | Assessment of Outcome | Adequate follow-up length | Adequacy of  follow-up |  |
| Braunstein 2015 | ☆ | ☆ | ☆ | ☆ | ☆ | ☆ |  | ☆ | 7 |
| Deville 2011 | ☆ | ☆ | ☆ | ☆ | ☆☆ | ☆ | ☆ | ☆ | 9 |
| Ishii 2017 | ☆ | ☆ | ☆ | ☆ | ☆ |  | ☆ |  | 7 |
| Link 2019 | ☆ | ☆ | ☆ | ☆ | ☆☆ | ☆ | ☆ |  | 8 |
| Mantini 2011 | ☆ | ☆ | ☆ | ☆ | ☆☆ | ☆ | ☆ | ☆ | 9 |
| McDonald 2014 | ☆ | ☆ | ☆ | ☆ | ☆ | ☆ | ☆ | ☆ | 8 |
| Moghanaki 2012 | ☆ | ☆ | ☆ | ☆ | ☆ | ☆ |  |  | 6 |
| Ramey 2017 | ☆ | ☆ | ☆ | ☆ | ☆ | ☆ |  |  | 6 |
| Song 2019 | ☆ | ☆ | ☆ | ☆ | ☆☆ | ☆ | ☆ |  | 8 |
| Waldstein 2017 | ☆ | ☆ | ☆ | ☆ |  | ☆ | ☆ | ☆ | 7 |

**Supplementary Figure 7. Funnel Plots for Main Outcome Comparisons**

**
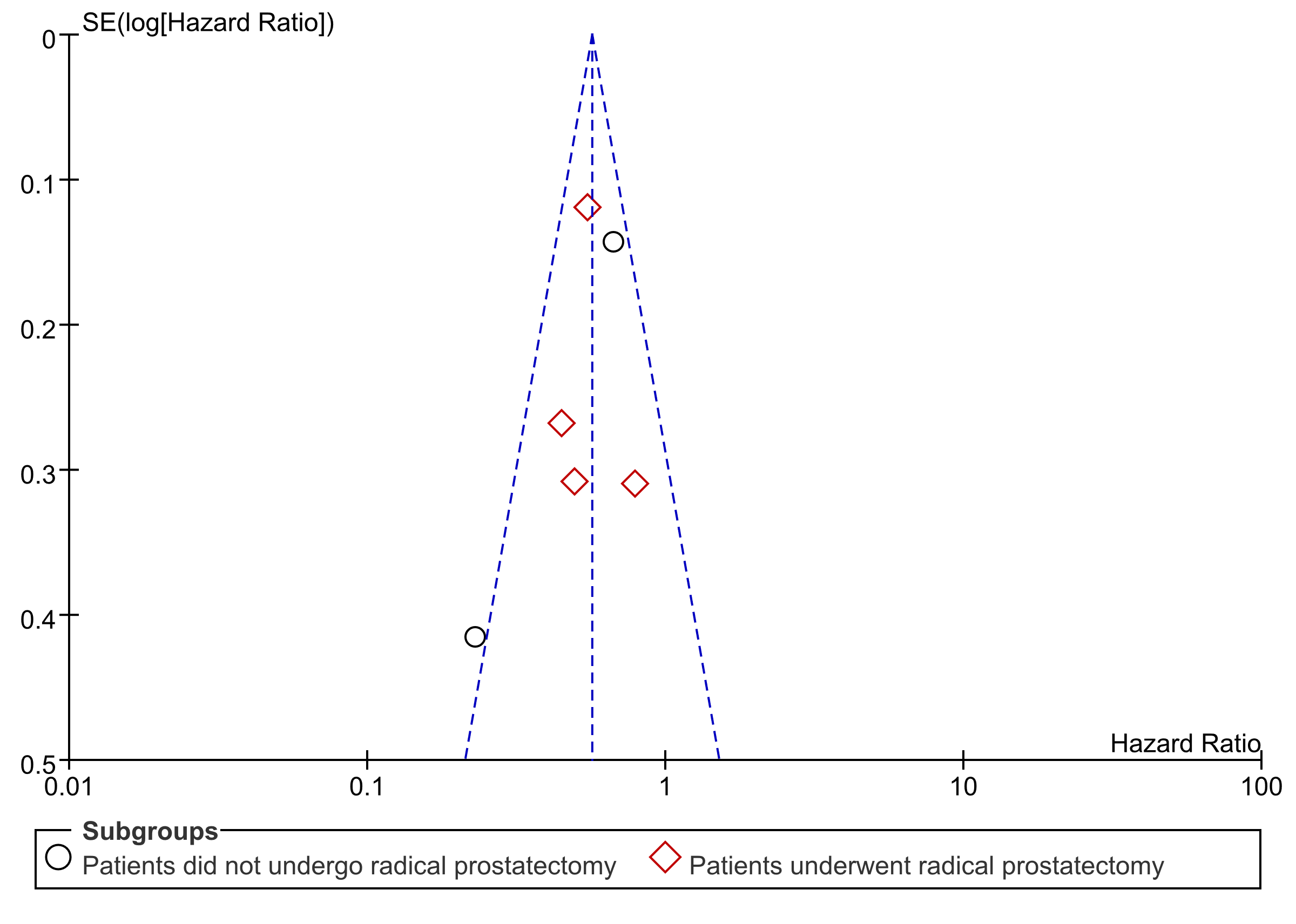
**

**Fig. 7A** **Funnel plot** **of the meta-analysis for BFFS.**


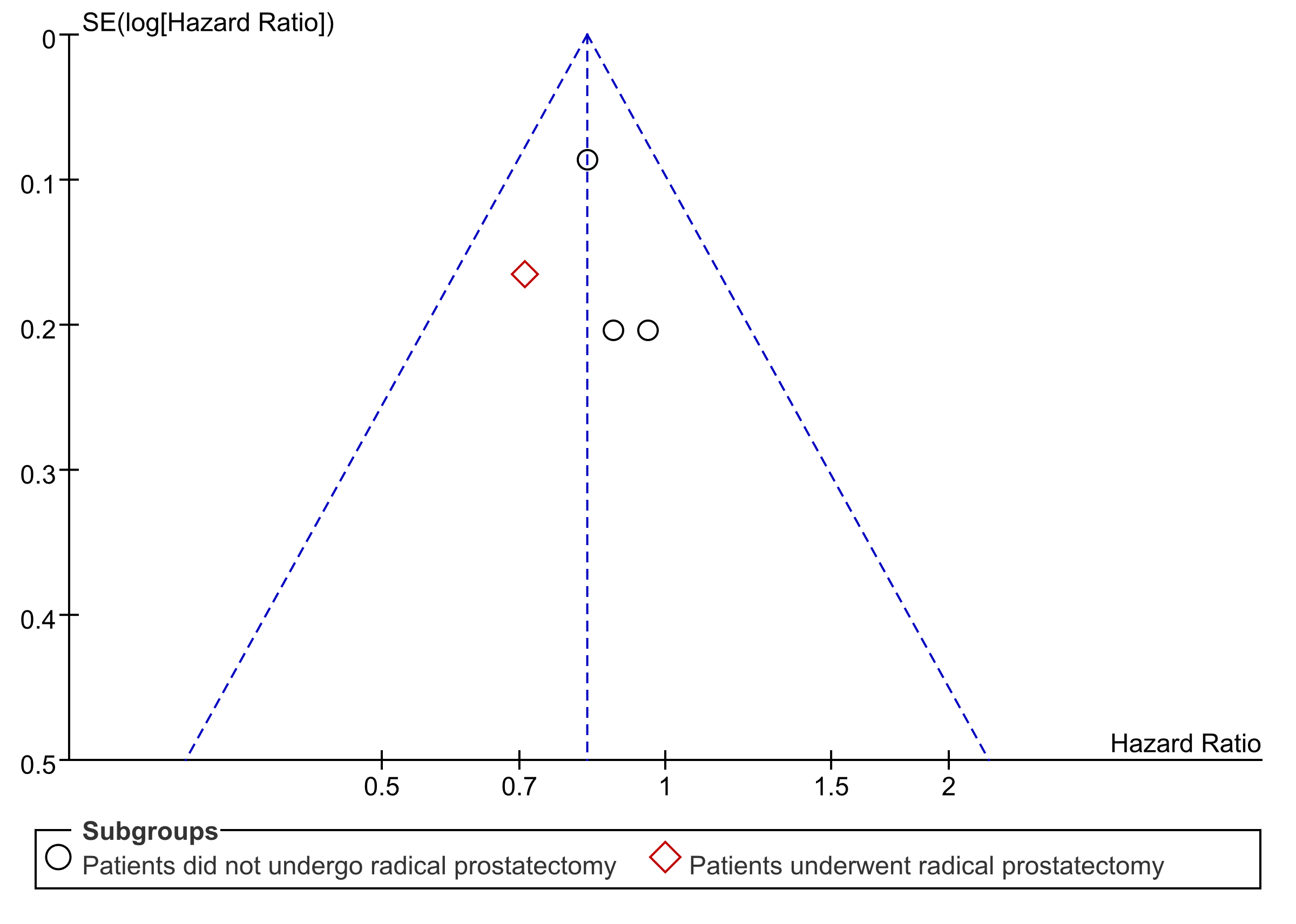


**Fig. 7B Funnel plot** **of the meta-analysis for PFS.**


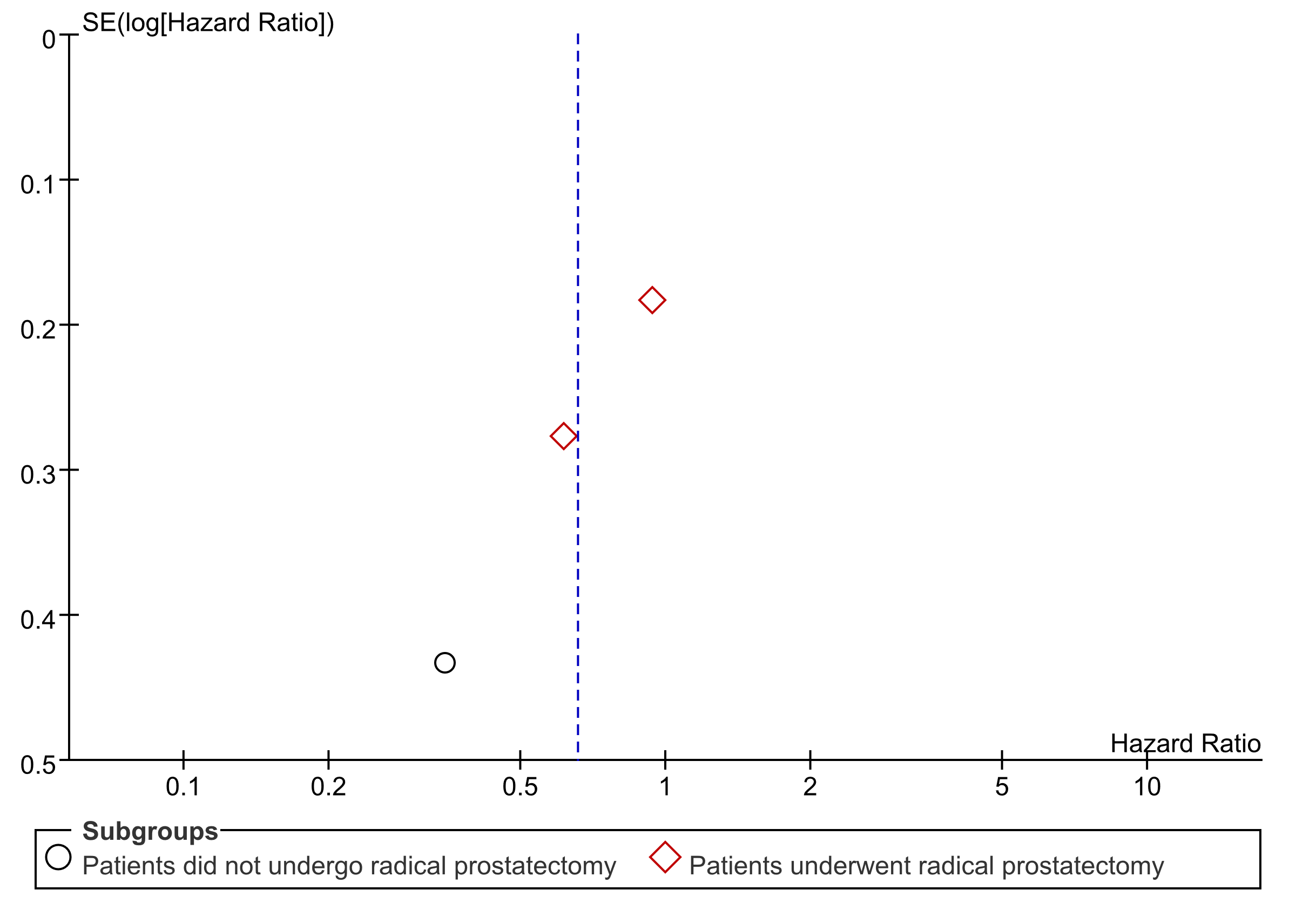


**Fig. 7C Funnel plot** **of the meta-analysis for DMFS.**


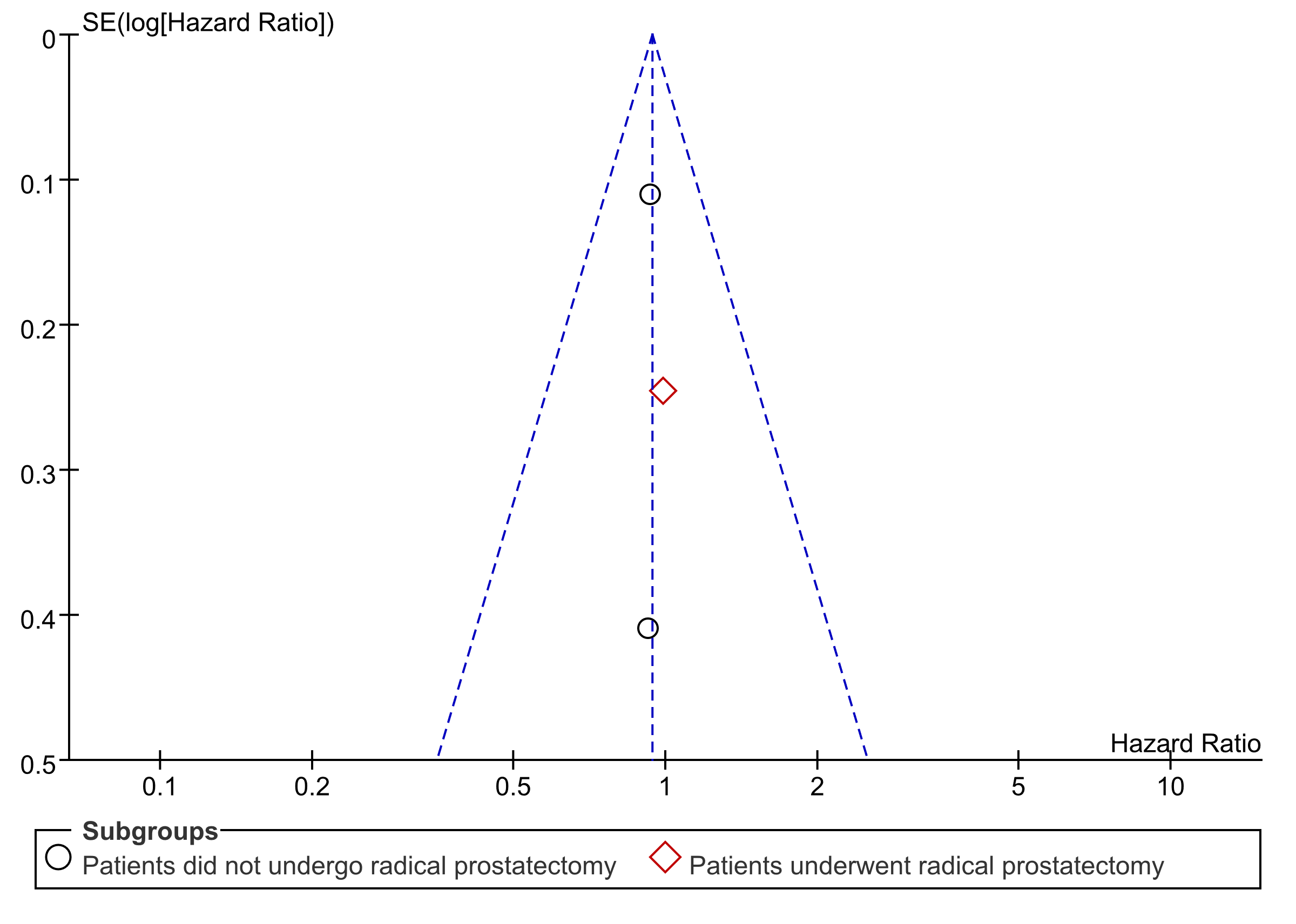


**Fig. 7D Funnel plot** **of the meta-analysis for OS.**

**
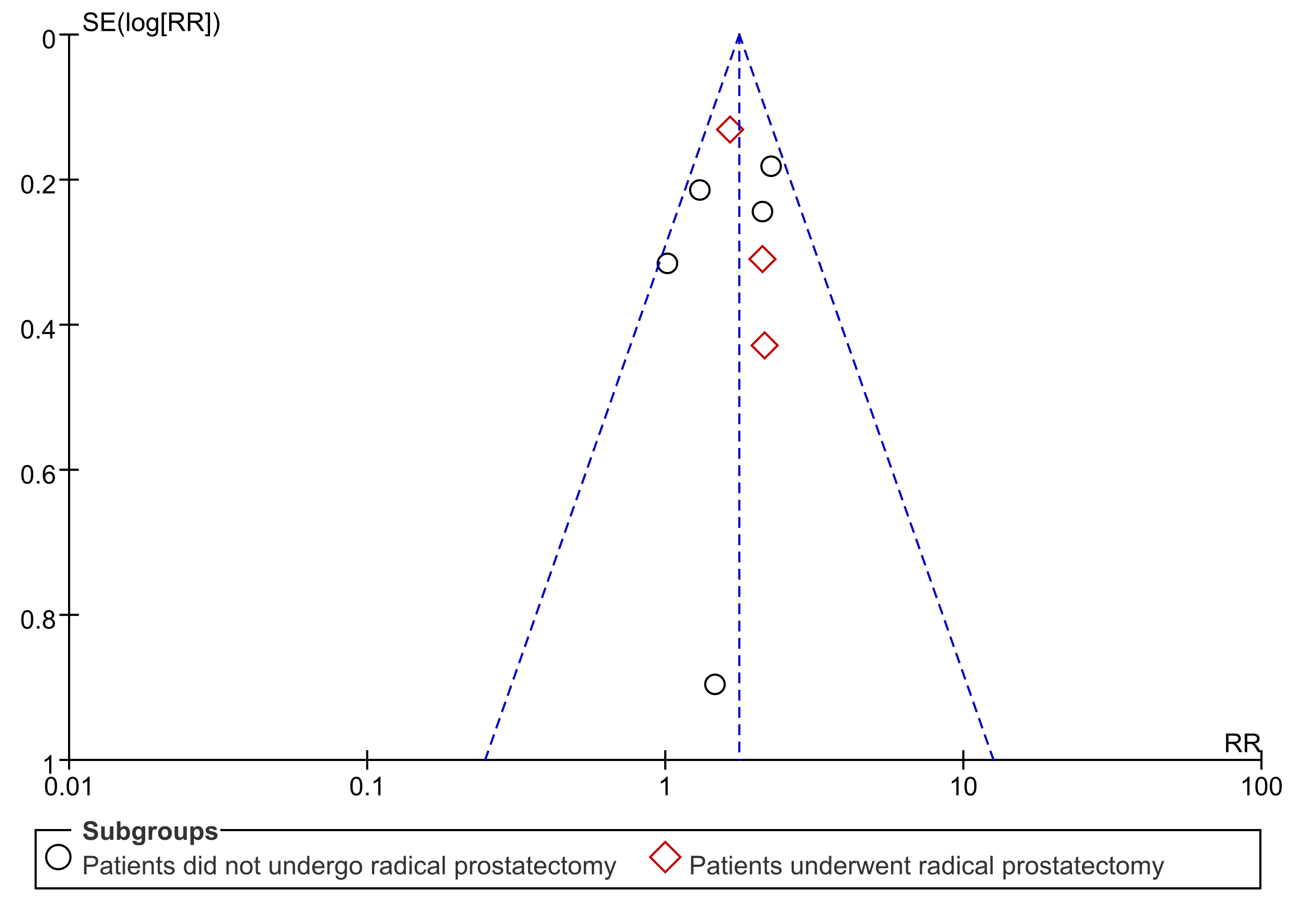
**

**Fig. 7E Funnel plot** **of the meta-analysis for acute GI.**

**
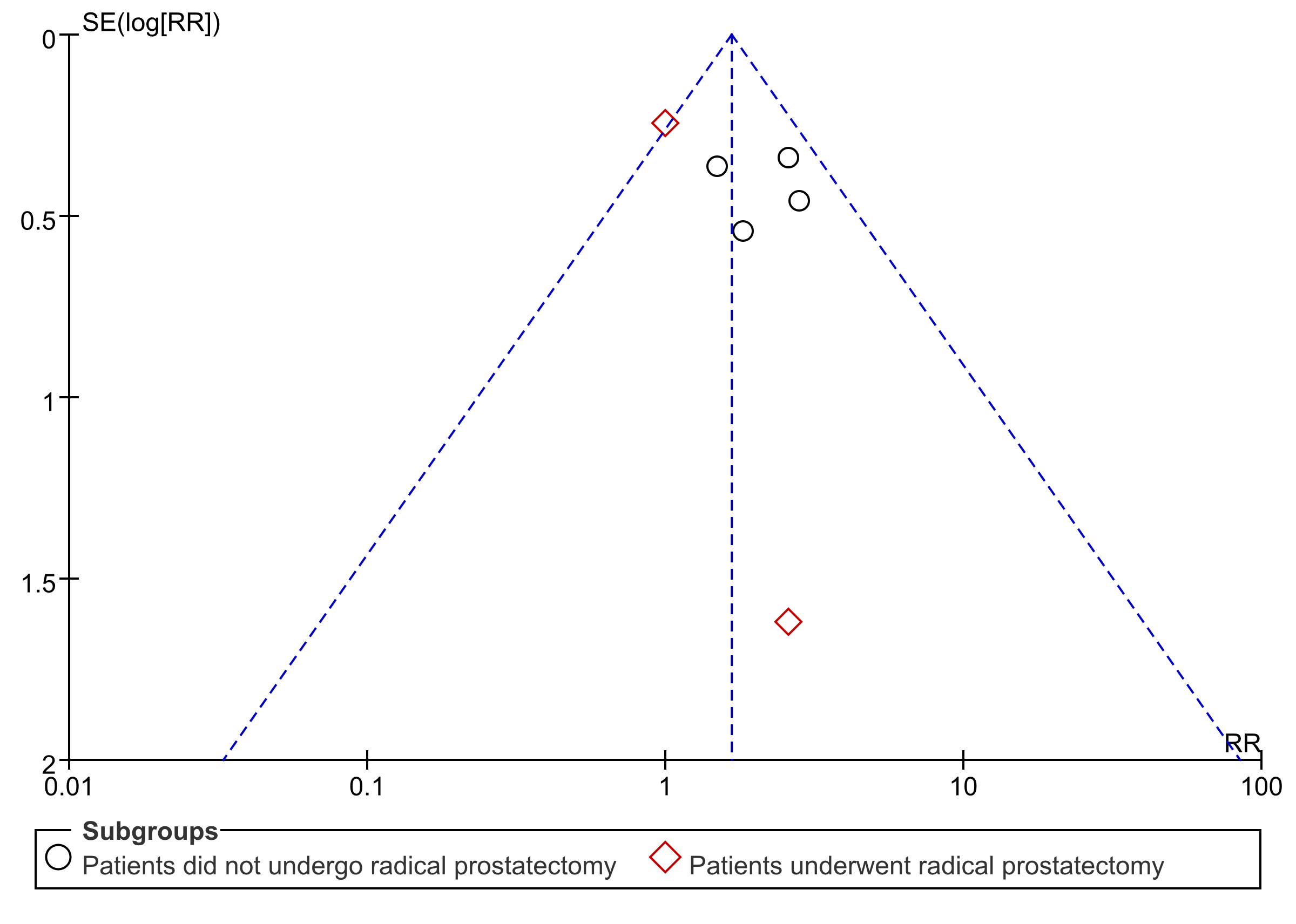
**

**Fig. 7F Funnel plot** **of the meta-analysis for late GI.**

**
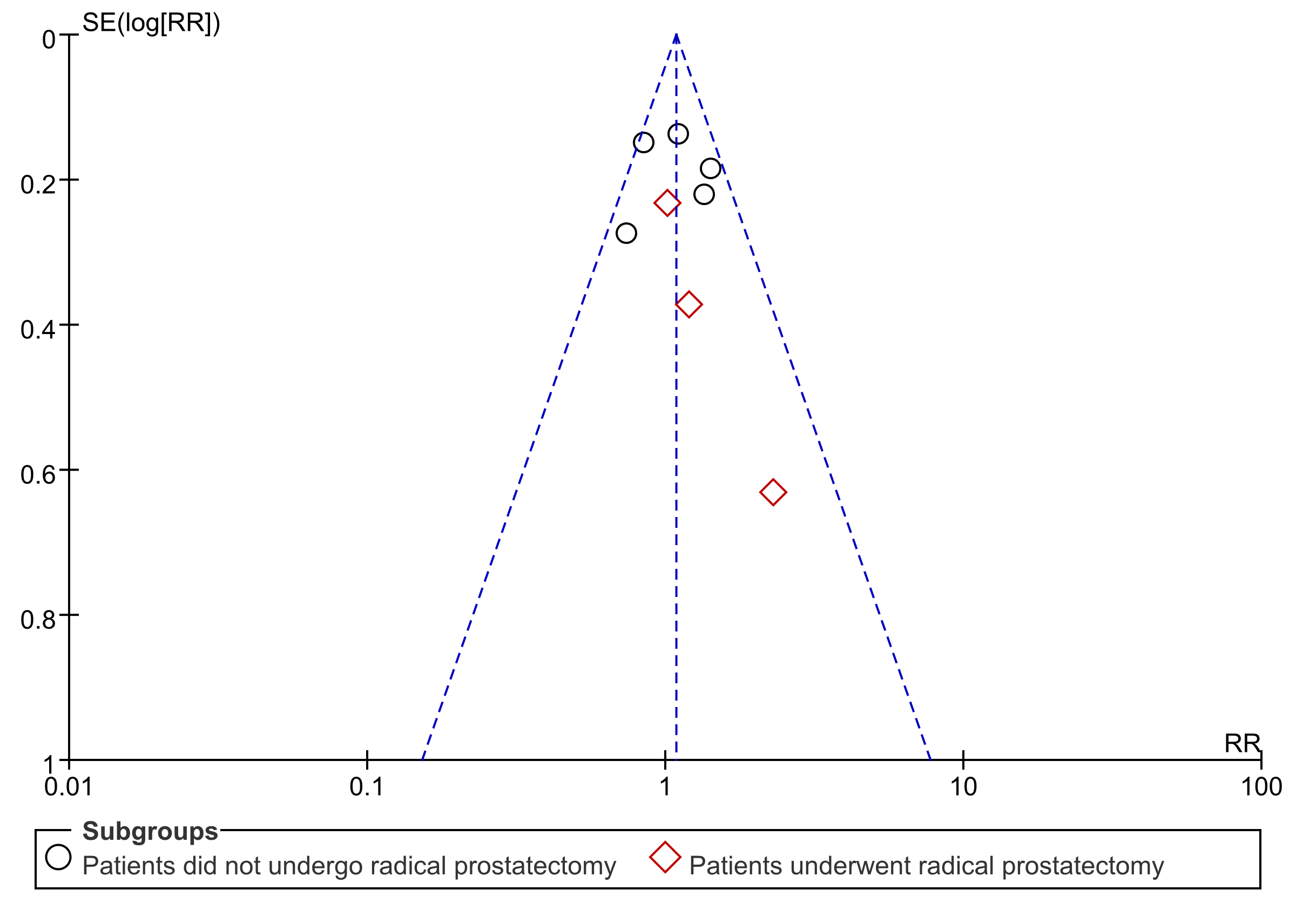
**

**Fig. 7G Funnel plot** **of the meta-analysis for acute GU.**

**
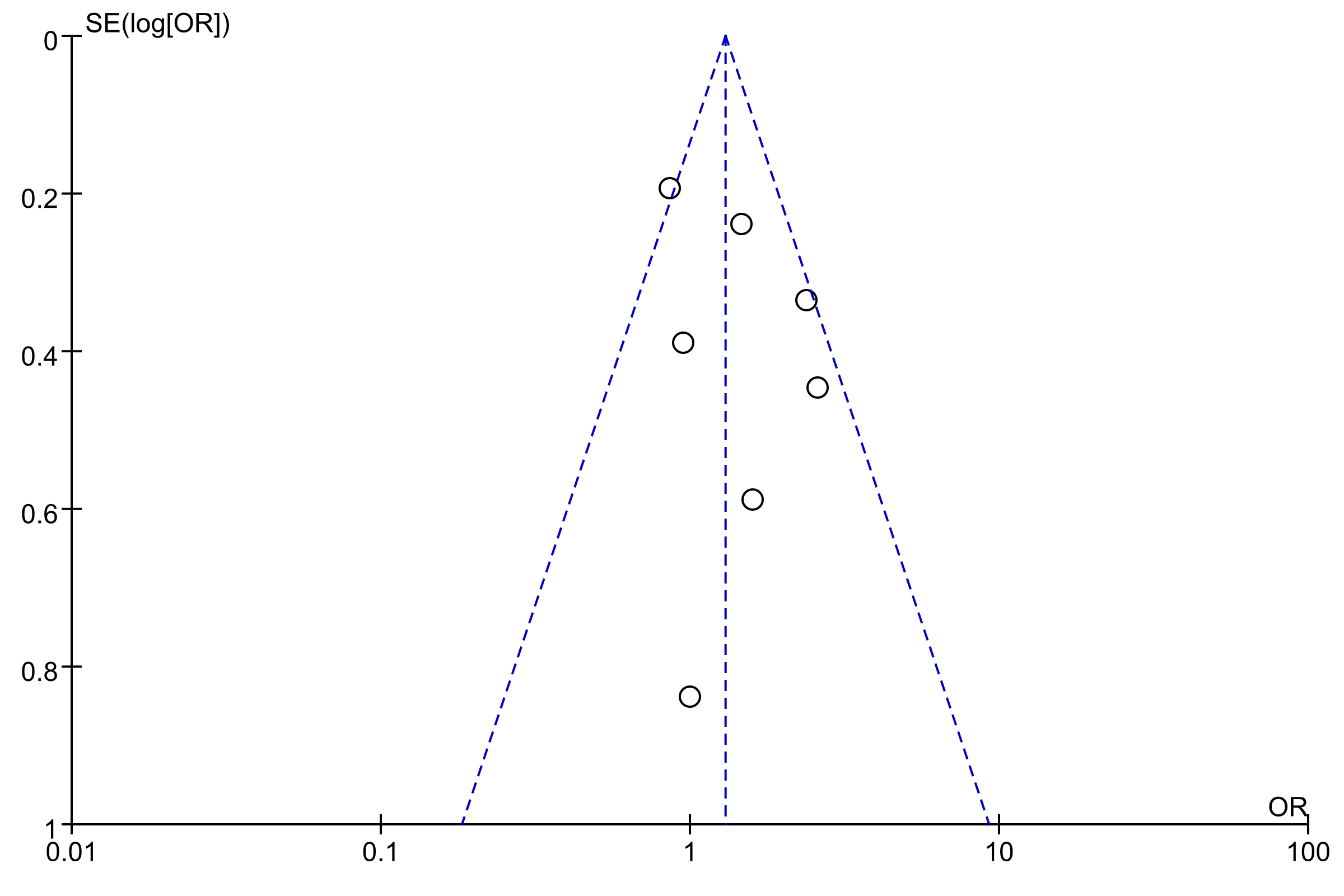
**

**Fig. 7H Funnel plot** **of the meta-analysis for late GU.**
